# Identification and Functional Characterization of Sugarcane Invertase Inhibitor (*ShINH1*): A Potential Candidate for Reducing Pre- and Post-harvest Loss of Sucrose in Sugarcane

**DOI:** 10.3389/fpls.2018.00598

**Published:** 2018-05-03

**Authors:** Suresha G. Shivalingamurthy, Raveendra Anangi, Sundaravelpandian Kalaipandian, Donna Glassop, Glenn F. King, Anne L. Rae

**Affiliations:** ^1^CSIRO Agriculture and Food, St Lucia, QLD, Australia; ^2^ICAR-Sugarcane Breeding Institute, Coimbatore, India; ^3^Division of Chemistry and Structural Biology, Institute for Molecular Bioscience, The University of Queensland, St Lucia, QLD, Australia

**Keywords:** INVINH proteins, heterologous expression, functional identity, sucrose accumulation, post-harvest deterioration, sugar yield

## Abstract

In sugarcane, invertase enzymes play a key role in sucrose accumulation and are also involved in futile reactions where sucrose is continuously degraded during the pre- and post-harvest period, thereby reducing sugar yield and recovery. Invertase inhibitor (INVINH) proteins play a key role in post-translation regulation of plant invertases through which sucrose hydrolysis is controlled. INVINH proteins are small (18 kDa) members of the pectin methylesterase inhibitor superfamily and they are moderately conserved across plants. In the present study, we identified two INVINH genes from sugarcane, *ShINH1* and *ShINH2*. *In silico* characterization of the encoded proteins revealed 43% sequence identity at the amino acid level, confirming the non-allelic nature of the proteins. The presence of putative signal peptide and subcellular targeting sequences revealed that ShINH1 and ShINH2 likely have apoplasmic and vacuolar localization, respectively. Experimental visualization of ShINH1–GFP revealed that ShINHI is indeed exported to the apoplast. Differential tissue-specific and developmental expression of *ShINH1* between leaf, stalk, flower and root suggest that it plays a role in controlling source-sink metabolic regulation during sucrose accumulation in sugarcane. *ShINH1* is expressed at relatively high levels in leaves and stalk compared to flowers and roots, and expression decreases significantly toward internodal maturity during stalk development. *ShINH1* is expressed at variable levels in flowers with no specific association to floral maturity. Production of recombinant ShINH1 enabled experimental validation of protein function under *in vitro* conditions. Recombinant ShINH1 potently inhibited acid invertase (IC_50_ 22.5 nM), making it a candidate for controlling pre- and post-harvest deterioration of sucrose in sugarcane. Our results indicate that *ShINH1* and *ShINH2* are likely to play a regulatory role in sucrose accumulation and contribute to the improvement of sugar yield and recovery in sugarcane.

## Introduction

Sugarcane (*Saccharum* spp.) is the major source of sucrose worldwide. Annually, 1,800 million tons of cane produced in an area of 27 Mha contributes ~80% of world sugar production (FAOSTAT, 2014). Sucrose metabolism is a complex process in sugarcane involving transfers between a number of compartments for synthesis, transport, and accumulation. The major source tissue for sucrose synthesis is mature leaves and sucrose is subsequently transported to sinks including growing points and the storage tissues of mature internodes where it may be further metabolized or accumulated. The net amount of sucrose accumulated in the storage parenchyma of the stalk depends on the balance between sucrose synthesis and breakdown activities (Moore, 1995). A remarkable feature of sugarcane is its capacity to store sucrose to about 50% of its dry weight. However, at crop maturity when the sucrose concentration in the sink tissues reaches saturation, the action of invertase enzymes affects sucrose stabilization, and reduces sucrose yield (Chandra et al., 2012). Also during the post-harvest period, activation of endogenous invertase enzymes leads to deterioration of sucrose in the cut cane and ultimately low sugar recovery (Solomon, 2009). Thus, sucrose breakdown by invertases causes a major economic loss to farmers and sugar processors. The rise in invertase activity and consequent loss of sucrose become increasingly problematic as the time gap between harvesting and milling increases. Singh et al. (2008) reported a 1.38- and 4.75-fold increase in invertase activity after 48 and 240 h of standing post-harvest, respectively, as compared to the initial activity in freshly cut cane. When milling of the cane cannot be completed within a day of cutting, other approaches are needed to preserve sucrose content. Suppression of invertase activity to stabilize sucrose content at cane maturity and after harvest has been investigated as a possible solution (Solomon, 2009).

Invertases [EC 3.2.1.26; β-fructosidase] are a family of enzymes known to perform diverse functions in plants in addition to hydrolysis of sucrose into glucose and fructose. In sugarcane, invertases are mainly involved in regulation of sucrose accumulation and plant development. (Moore, 1995; Zhu et al., 1997). Sucrose unloaded from the phloem in the sugarcane stalk is transported to three different cellular compartments: the apoplastic space (cell wall), cytoplasm, and vacuole (Bieleski, 1960; Hatch and Glasziou, 1963; Sacher et al., 1963; Ma et al., 2000). Each compartment has a specific invertase isoform: an apoplastic space located cell wall invertase (CWI), a vacuolar located acid invertase (VAI) also termed a soluble acid invertase (SAI), and a cytoplasmic located neutral invertase (NI) (Ma et al., 2000). Sucrose accumulated in sugarcane stalks is probably resynthesized from the hydrolysis products of translocated sucrose following breakdown by invertase enzymes (Hatch and Glasziou, 1963; Ma et al., 2000). Sucrose synthase (SuSy) and invertases are involved in futile cycling during the process of sugar transport between cytoplasm, vacuole and cell wall, which is sometimes energetically wasteful (Botha et al., 2001) due to continuing sucrose hydrolysis and resynthesis. In this way sucrose/hexose interchange determines net sucrose content in sugarcane juice. The major function of invertases is to maintain high hexose concentration, and the hydrolysis of sucrose in the vacuole and in the intracellular space affects the sucrose yield in sugarcane (Whittaker and Botha, 1997; Wu and Birch, 2007; Wang et al., 2013). Because most previous studies have focused on SAI, the role of other sugarcane invertase isoforms in regulation of sucrose accumulation is poorly understood. (Ma et al., 2000; Botha et al., 2001; Chandra et al., 2012). Thus, there is a need for further information on sugarcane invertases before specific strategies for manipulation can be defined.

Invertase has been a target for molecular manipulation to alter carbohydrate accumulation in a number of plants, including *Arabidopsis* (von Schaewen et al., 1990), tobacco (Sonnewald et al., 1991), tomato (Ohyama et al., 1995), potato (Bussis et al., 1997), and carrot (Tang et al., 1999). There have been efforts to control invertase enzymes (both SAI and NI) in sugarcane through transgenesis but despite producing successful transgenic events, there was no significant increase in overall sucrose yield (Ma et al., 2000; Botha et al., 2001; Rossouw et al., 2007). This may be due to regulatory feedback between sink and source during sucrose accumulation (McCormick et al., 2006, 2009; Chandra et al., 2011). In the light of these results, novel avenues are needed to manipulate plant invertases and study the possible effects on sucrose content in sugarcane. Post-translational silencing of invertase enzymes using specific inhibitor proteins is an approach that has not previously been tested.

The extent of proteolytic degradation of invertases depends on non-covalent complex formation with invertase inhibitor (INVINH) proteins (Rausch and Greiner, 2004). The first plant INVINH was discovered in potato, with homologs subsequently identified in red beet, sugar beet, sweet potato and maize (Schwimmer et al., 1961; Pressey, 1968; Jaynes and Nelson, 1971; Kursanov et al., 1971). The first gene encoding an INVINH protein was cloned from tobacco (*Nicotiana tabacum*), and the recombinant protein was shown to be an effective inhibitor of invertase activity *in vitro* (Greiner et al., 1998). Overexpression of the tobacco gene in potato reduced invertase activity *in vivo* and suppressed cold-induced sweetening of tubers (Greiner et al., 1999). Further studies showed the attenuation of apoplastic INVINH activity by sucrose. The results suggested that sink strength and partitioning may be controlled by a regulatory mechanism involving three components: invertase, inhibitor, and sucrose (Sander et al., 1996; Krausgrill et al., 1998). The first monocot INVINH gene was cloned from maize, where it was found to be expressed during early kernel development and involved in regulation of endosperm formation by inhibiting apoplasmic invertase (Bate et al., 2004).

Manipulation of specific invertase inhibitor genes by overexpression or silencing has been tested in a range of plants as a strategy for modulating invertase activity. In addition to the intended target of sucrose metabolism, these approaches can also affect other physiological processes including seed development, leaf senescence, and responses to both biotic and abiotic stresses. For example, Jin et al. (2009) showed that inhibition of the CWI in tomato by the INVINH1 inhibitor regulated leaf senescence and the development of seeds and fruits. Similarly, in soybean, silencing of the GmCIF1 inhibitor lead to increased activity of CWI which modified sucrose metabolism and sink strength and enhanced the maturation of seeds (Tang et al., 2017). Embryo growth may also be impacted by modulation of invertase inhibitors, since an important role for an endosperm-expressed INVINH in *Arabidopsis* was recently demonstrated (Zuma et al., 2018). It has been shown that invertases are involved in regulation of stomatal opening (Ni, 2012) and ectopic expression of a tobacco vacuolar invertase inhibitor in *Arabidopsis* changed stomatal function resulting in improved drought tolerance (Chen et al., 2016). Enhanced cold tolerance in tomato was achieved through silencing of the INVINH1 inhibitor which caused an increase in the activity of CWI (Xu et al., 2017).

Given the potential of INVINH proteins in controlling invertase activity, we performed the first exploration of the sugarcane genome for INVINH genes. This led to identification, cloning and characterization of two sugarcane INVINH genes, *ShINH1* and *ShINH2*. We completed a functional characterization of *ShINH1*, demonstrating temporal and spatial expression, subcellular localization in a heterologous system, and invertase inhibitory activity of recombinant ShINH1. This is the first study where the functional identity of a sugarcane INVINH gene has been established by evaluating the inhibitory activity against invertase. The results of this study will underpin future efforts to control invertase-induced losses of sucrose in post-harvest sugarcane.

## Materials and methods

### Plant material and growth conditions

The *Saccharum* hybrid cultivar Q208 was used for all experiments. This variety was selected for experiments due to its wide adaptability and highest tonnage among the cultivated varieties in Australia (https://sugarresearch.com.au).

Individual plantlets, arising from single bud setts of *Saccharum* hybrid Q208 were germinated in vermiculite, and were transplanted to 8 L pots containing Searles Peat 80 Mix (Searles, Australia). Setts were germinated monthly to produce a series of plants with different ages from 5 to 8 months (March, 5th month: 150 DAP); April, 6th month: 180 DAP; May, 7th month: 210 DAP and June, 8th month: 240 DAP). Plant growth conditions consisted of 14 h light (500 μmol photons m^−2^s^−1^) conditions at 30°C/55% temperature and humidity, respectively, and 10 h dark conditions at 24°C/65%; with daily watering. Internodes were numbered according to previously published research whereby the first top leaf with a visible dewlap is attached to internode 1 (Kuijper, 1915; Moore, 1987). During harvesting, internodes 4 (young immature), 8 (moderately mature), and 13 (fully mature) were cut into 1 cm cubes and immediately frozen in liquid N_2_ and then stored at −80°C. Frozen samples were ground into a fine powder using a ball mill (MM400, Retsch, Germany) at 30 Hz for 2 min. The powdered samples were dried using a vacuum freeze drier (Christ Alpha 1-4 LSC, Germany) and kept at room temperature until use.

For measurement of gene expression during flower development (240 DAP), flowers of cultivar Q208 from the Sugar Research Australia Experiment Station at Meringa (Gordonvale, Queensland) were collected. Three flower stages were selected depending on the maturity of inflorescence. Normally in plants which showed staggered flowering, three stages were assigned: stage 1 (young flowers), stage 2 (moderately mature), and stage 3 (fully mature flower) (Figure S1). These flowers were submerged in RNAlater^TM^ solution (Ambion, Austin, TX) and transported to Brisbane on the same day where they were stored at 4°C. Samples were snap frozen in liquid nitrogen and powdered before extraction of RNA.

For tissue-specific gene expression of *ShINH1*, samples of stalk, leaf, flower, and root from mature plants (10 months old) were collected and prepared as described earlier.

### RNA extraction

Total RNA was isolated from tissue samples using an RNeasy Plant Mini Kit (including DNase digestion to remove remaining DNA) (QIAGEN, Chadstone, VIC, Australia) according to the manufacturer's directions. Briefly, 100 mg of powdered tissue materials were suspended in 450 μl RLT buffer (supplied with kit) and vortexed vigorously. Resulting tissue lysate was transferred to a spin column (Qiashredder) placed in 2 ml collection tube and centrifuged at 13,000 rpm for 2 min. To the supernatant, 500 μl of ethanol (100%) was added and mixed immediately by pipetting. Resulting cleared lysate was transferred to new spin column and centrifuged at 10,000 rpm for 30 s. RNA bound spin column was washed twice with RW1 buffer (supplied with kit) and eluted with RNAse free water. All RNA samples were stored at −80°C until required.

### Gene isolation and cloning

To identify INVINH genes from sugarcane, *Zea mays* (NM_001157609) and *Nicotiana tabacum* (Y12806) INVINH sequences were used in BLASTn searches against sugarcane genome scaffolds available from the CSIRO sugarcane genome sequencing project using CLC Genomics Workbench (www.qiagenbioinformatics.com/products/clc-genomics-workbench/). We identified three homologs of the *Z. mays* INVINH gene but none of the tobacco INVINH gene (Figure S2). Primers were designed in different combinations to match the start and stop codon regions as well as 5′ and 3′ UTR region of the genomic sequences (Table 1). Oligonucleotides were custom synthesized by GeneWorks, Australia. Primer pairs were tested for their ability to amplify *ShINH* genes. Full-length sequences of two sugarcane INVINH genes were amplified (*ShINH1* and *ShINH2*) from cDNA (0.5 μg) synthesized from RNA isolated from internodal tissues, using a standard PCR amplification protocol with high-fidelity Platinum Taq Polymerase (Thermo Fisher Scientific, USA) (Figure S3). Amplified products were cloned into the pGEM-T Easy vector (Promega, Madison, WI, USA), then *E. coli* DH5α cells transformed with recombinant plasmid were selected based on ampicillin resistance. Plasmids were isolated from confirmed colonies and restriction analysis performed using *Eco*RI to confirm the presence of a cloned gene (Figure S3). Recombinant plasmids (pGEM-T Easy-*ShINH1*/*ShINH2*) were sequenced using the dideoxy chain termination method (Sanger et al., 1977) using T7 and SP6 primers (Australian Genome Research Facility, The University of Queensland, Brisbane, Australia).

**Table 1:** Sequences of primers used for gene isolation, transcript expression assessment, vector construction for GFP localization, and recombinant protein expression.

**Primer #**	**Orientation**	**Use**	**Sequence (5′-3′)**
1	Forward	INH1 gene isolation	ATGAAGCTTCTGCAAGCTCTG
2	Reverse	INH1 gene isolation	CTACAGCGCCTCCGTTACAGA
3	Forward	INH2 gene isolation	ATGAAGCTAGTCTGCTCCGTG
4	Reverse	INH2 gene isolation	TTAATCACTAATCTTTGGCCT
5	Forward	INH1 qRTPCR	CGTCCAACGCTTCCGTCTTA
6	Reverse	INH1 qRTPCR	GTCGGCCTGGAAGAACTTGA
7	Forward	ADF qRTPCR	CTACTACTGTGGATTTGTACGCCATTATAG
8	Reverse	ADF qRTPCR	GGACCTTTTTTACACAGCAACAAAC
9	Forward	INH1-GFP vector construct preparation	TATAACTAGTATGAAGCTTCTGCAAGCTCTG
10	Reverse	INH1-GFP vector construct preparation	GCGGCCGCTTACTACAGCGCCTCCGTTACAGA
11	Forward	PLIC INH1 expression vector construct preparation	TATATGGTACCGAGAACCTGTACTTCCAATC
12	Reverse	PLIC INH1 expression vector construct preparation	ACATAGAGCTCTTACTACAGCGCCTCCGTTAC

### *In silico* characterization

Nucleotide sequences of the *ShINH1* and *ShINH2* clones and their deduced amino acid sequences were used to identify the genes using BLAST programs at NCBI. Prediction of putative signal peptides was carried out using PrediSi (Hiller et al., 2004) and SignalP (Petersen et al., 2011) programs, while subcellular localization was predicted using PSORT (www.genscript.com/wolf-psort.html). Multiple sequence and pairwise alignments were performed using CLC Genomics Workbench. A phylogenetic tree was constructed with the neighbor-joining method using Mega 7.0 software (Kumar et al., 2016). Secondary structure prediction of ShINH1 was carried out using PSIPRED (http://bioinf.cs.ucl.ac.uk/psipred/) (Jones, 1999).

### GFP localization

To examine subcellular localization of ShINH1, wheat leaf and onion epidermal cells were transformed with Ubi1GFP–ShINH1 or Ubi1GFP gene constructs through particle bombardment using the method described by Xue (2002). Leaves of 1-month-old wheat plants grown in a glasshouse under controlled conditions and fresh onion bulbs purchased locally were used. Newly expanded wheat leaves and onion epidermal tissue layers were freshly excised and placed in the center of a Petri dish on two layers of Whatman No. 1 filter paper saturated with a solution of 100 mM sucrose, 5 mM sodium phosphate, pH 7.0. The ZmUbi1 promoter-driven GFP constructs with or without ShINH1 fusion (Ubi1GFP–ShINH1 or Ubi1GFP) were constructed according to Xue (2002) and the UbiGFP without insert was used as a control (Figure 3A). Plasmid DNA was precipitated onto gold particles (1 μm) and bombarded into wheat leaves and onion epidermal layers twice to increase the number of transformed cells in the tissues. The particle inflow gun was used at a pressure of 2,100 kPa and a vacuum of 28 mmHg (Patel et al., 2000). The bombarded tissues were kept at 20°C for 24 h. Bombarded tissues were examined using fluorescence (Leica MZ16FA) and confocal (Zeiss Axiovert-200 with LSM 710 Meta Confocal Scanner) microscopy with excitation/emission of 480/510 and 470/525 nm, respectively.

### Expression analysis using real-time quantitative PCR (RT-qPCR)

For RT-qPCR analysis, cDNA was synthesized using a QIAGEN cDNA synthesis kit. Forward and reverse primers for *ShINH1* gene were designed from the transcript sequence. A gene encoding actin depolymerising factor (ADF, GenBank CO373080) was used as the endogenous control (Casu et al., 2007). The expression level of *ShINH1* was quantified from cDNA samples using a ViiATM 7 system (Applied Biosystems) and SYBR Green PCR Master Mix (Applied Biosystems) according to the manufacturer's instructions. Relative quantitation of mRNA levels was as described by Shaw et al. (2009).

### Production and purification of recombinant ShINH1

The pLic-MBP expression vector was used to produce recombinant *ShINH1* following the method described by Anangi et al. (2012) with some modifications. This vector encodes a MalE signal sequence for periplasmic export, a His_6_ affinity tag, a maltose binding protein (MBP) fusion tag to aid solubility, and a TEV protease recognition site directly preceding the ShINH1-coding region (Figure 5A). The plasmid encoding His_6_-MBP-ShINH1 was transformed into *E. coli* strain BL21 for recombinant protein production. For protein production, the pre-inoculum was grown in LB medium overnight at 37°C with shaking (180 rpm). The overnight cultures were diluted to 1 L with fresh LB medium and growth continued at 37°C. Expression of *ShINH1* was induced with 1 mM IPTG when the OD_600_ reached 0.8. Cultures were then grown overnight at 18°C with shaking at 180 rpm, after which bacteria were pelleted by centrifugation at 5,000 *g* for 20 min at 4°C. Three approaches were initially tested for recovery of the soluble His_6_-MBP-ShINH1 fusion protein: (i) cells were lysed using a high-pressure cell disruptor (TS Series Benchtop model, Constant Systems Ltd, UK) in equilibration buffer (20 mM Tris, 300 mM NaCl, 10% glycerol, pH 8.0); (ii) sonication (in the equilibration buffer); and (iii) extraction of the periplasmic fraction by osmotic shock (30 mM Tris, 20% sucrose, pH 8.0 and ice-cold water). The soluble His_6_-MBP-ShINH1 fusion protein (buffered in 20 mM Tris, 300 mM NaCl, 10% glycerol, 5 mM imidazole, pH 8.0) was captured by passing the cell extract by gravity flow through a Ni-NTA Superflow resin (QIAGEN, Valencia, CA, USA). Unbound protein was eluted with equilibration buffer containing 15 mM imidazole, then the fusion protein was then eluted with 25–500 mM imidazole. All fractions were examined for the presence of target protein using SDS-PAGE (Laemmli, 1970). Protein samples were pooled and concentrated using a 30-kDa cutoff centrifugal filter (Amicon). The fusion partner (His_6_-MBP) was removed by adding reduced and oxidized glutathione (3.0 and 0.3 mM, respectively) to activate TEV protease. TEV protease was added (approximately 40 μg per mg of His_6_-MBP-ShINH1), and the reaction was allowed to proceed at room temperature for 12 h. The liberated ShINH1 was then separated from TEV protease, His_6_-MBP, and uncleaved fusion protein using gel filtration chromatography (Sephacryl HiPrep S-200; GE Healthcare). The concentration of recombinant ShINH1 was determined from A_280_ absorbance measured using a NanoDrop spectrophotometer.

### Circular dichroism (CD)

Approximately 250 μg of recombinant ShINH1 (from gel filtration chromatography) was dialyzed against 10 mM Tris and 100 mM NaF overnight at 4°C. The dialyzed ShINH1 was then concentrated using a 10-kDa cut-off centrifugal filter (Amicon). CD spectra (190–250 nm range, 25°C) were recorded from a 63.3 μM sample of ShINH1 in a 0.1 mm cuvette using a Jasco J-180 spectropolarimeter.

### Invertase activity assay

A commercial invertase assay kit employing yeast invertase (Cat.MAK118, Sigma Aldrich, USA) was used as per the manufacturer's protocol to examine the potency of recombinant ShINH1. Briefly, 40 μl of reaction volume containing different concentrations of ShINH1 (100–500 nM) were pre-incubated in a 96-well plate with commercial acid invertase at 37°C for 30 min. Glucose standards (40 μl volume, 0–100 μM glucose) were added to separate wells of the plate. The same volume of reaction buffer was used as the assay blank in separate wells. Substrate was added to each well (5 μl of 20 mM sucrose) followed by incubation for 20 min at room temperature. After incubation, the reaction mixture containing 95 μl of reaction buffer, 1 μl of enzyme mix and 1 μl of dye reagent (all supplied with the kit) was prepared and 90 μl of reaction mix was added to each of the blank, sample, and standard wells followed by incubation for 20 min at room temperature in darkness. After incubation, absorbance was recorded at 570 nm using a microplate reader and the amount of glucose liberated was calculated from the glucose standard curve. The specific activity of enzyme was calculated and expressed as μmoles of glucose formed per milligram of protein per minute. The concentration of ShINH1 required for 50% inhibition of enzyme activity (IC_50_) was calculated by plotting nonlinear regression dose-response curve of Hill equation using GraphPad Prism 7.04 software.

### Statistical analysis

The analyses of gene expression and invertase enzyme activity were presented as means ± SDs of three biological replicates. The data were analyzed with Duncan's multiple range test using Statistical Analysis system (SAS) software version 9.2 (www.iasri.res.in/sscnars/). *P* < 0.01 was considered to be statistically significant.

## Results

### Identification and sequence characterization of *ShINH1* and *ShINH2*

Putative INVINH genes were identified in a sugarcane genome database assembled using short-read sequences from variety R570 (Aitken et al., 2016), covering over 97% of transcribed unigenes. Although both maize (NM001157609) and tobacco (Y12805 & Y12806) INVINH genes were used as query sequences, only the maize sequence aligned to sugarcane scaffolds, with three regions of homology identified (Figure S2). From these three genomic sequences, scaffold 413473 contained the longest alignment (640 bp); this sequence was designated *ShINH2*, and used directly for primer design and subsequent gene isolation. In contrast, scaffolds 2944351 and 762782 contained a distinct sequence that aligned to an uncharacterized sorghum genomic sequence of 531 bp, and this was designated *ShINH1*. Primers designed for the ORF regions amplified products of 531 and 576 bp for *ShINH1* and *ShINH2*, respectively (Figure S3). *ShINH1* and *ShINH2* code for putative proteins of 177 and 192 amino acid residues with molecular masses of 18.2 and 20.0 kDa, respectively (Figure S4). ShINH1 and ShINH2 are only 43.5% identical at the amino acid level, suggesting that they are not allelic forms but encode different INVINH proteins. The difference in protein characteristics between ShINH1 and ShINH2 are depicted in Table 2.

**Table 2:** Characteristics of ShINH1 and ShINH2 proteins.

**Feature**	**ShINH1**	**ShINH2**
Signal peptide (PREDISI)	1–21aa	1–19aa
Amino acids	177aa	192aa
Molecular weight	18.17 kDa	19.97 kDa
Isoelectric point	8.91	4.88
Localization signal (PSORT)	Apoplasmic	Vacuolar

**Figure 1:**
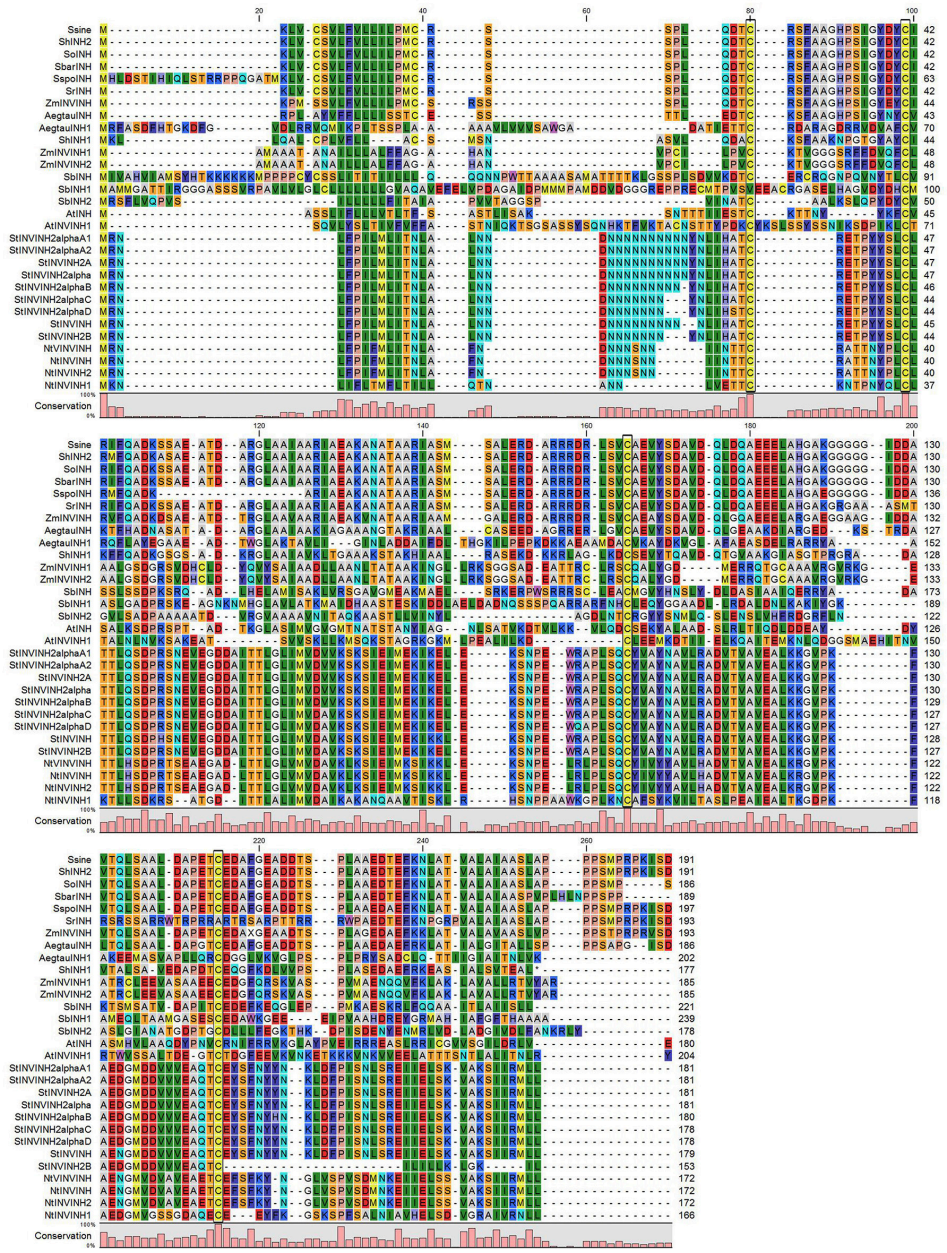
Comparison of ShINH1 & ShINH2 with plant invertase inhibitor-like proteins. Multiple sequence alignment of deduced amino acid sequences of plant invertase inhibitor (INVINH) proteins from *S. sinense* (SsineINH, KP997206); *Saccharum* hybrid (ShINH2); *S. officinarum* (SoINH, KP997207); *S. barberi* (SbarINH, KU057162); *S. spontaneum* (SspoINH, KP844455); *S. robustum* (SrINH, KP055631), *Zea mays* (ZmIN VINH, EU969422); *Aegilops tautschi* (AegtauINH, XM_020320985; AegtauINH1, XM_020311699); *Saccharum* hybrid, ShINH1); *Sorghum bicolor* (SbINH, XM_002453079; SbINH1, XM_002453080; SbINH2, XM_002452686), *Zea mays* (INVINH1, EU952678; INVINH2; EU960562); *Arabidopsis thaliana* (AtINH, Y12807; AtINVINH1, DQ056716); *Solanum tuberosum* (StINVINH2α, KJ788176; StINVINH2αB, FJ810207; StINVINH2αC, FJ810208; StINVINH2αD, FJ810209; StINVINH, JQ269669; StINVINH2B, GU321342), and *Nicotiana tobacum* (NtVINVINH, AY145781; NtINVINH, AY594179; NtINVINH2, Y12805; NtINVINH1, Y12806). Conserved Cys residues are indicated by boxes.

The deduced ShINH1 and ShINH2 protein sequences include putative signal peptides of 21 and 19 residues resulting in mature proteins of 15.8 and 17.0 kDa, respectively (Figure S5). Analysis of subcellular localization signals revealed that ShINH1 and ShINH2 are likely targeted to the extracellular space and vacuole, respectively (Figure S6). Alignment of the deduced sequences of ShINH1 and ShINH2 with other INVINH proteins from monocots and dicots revealed conservation of the four Cys residues that are a characteristic feature of all known plant INVINH proteins (Figure 1). Pairwise comparison of ShINH1 and ShINH2 with other plant INVINH proteins revealed that they cluster separately with plant INVINH1 and INVINH2 homologs, respectively. ShINH1 is most similar to monocot INVINH proteins, including *Saccharum* hybrid *SoINVInh1* (97.2%), *Sorghum bicolor* SbINHCWI/VAI (92.7%), *Z. mays* ZmINHCWI/VAI (85.9%), and *Setaria indica* SiPMEI (74.7%), and has less than 24% identity with dicot INVINH proteins (Figure S7). ShINH2 is more analogous to wild *Saccharum* INVINH proteins with highest similarity of 99% to *S. sinesse* (SsineINH), 95.8% to *S. officinarum* (SoINH), 92.3% to *S. barberi* (SbarINH), 82.1% to *S. spontaneum* (SspoINH), and 79.8% to *S. robustum* (SrINH), and in contrast has not more than 21% identity with dicot INVINH proteins.

**Figure 2:**
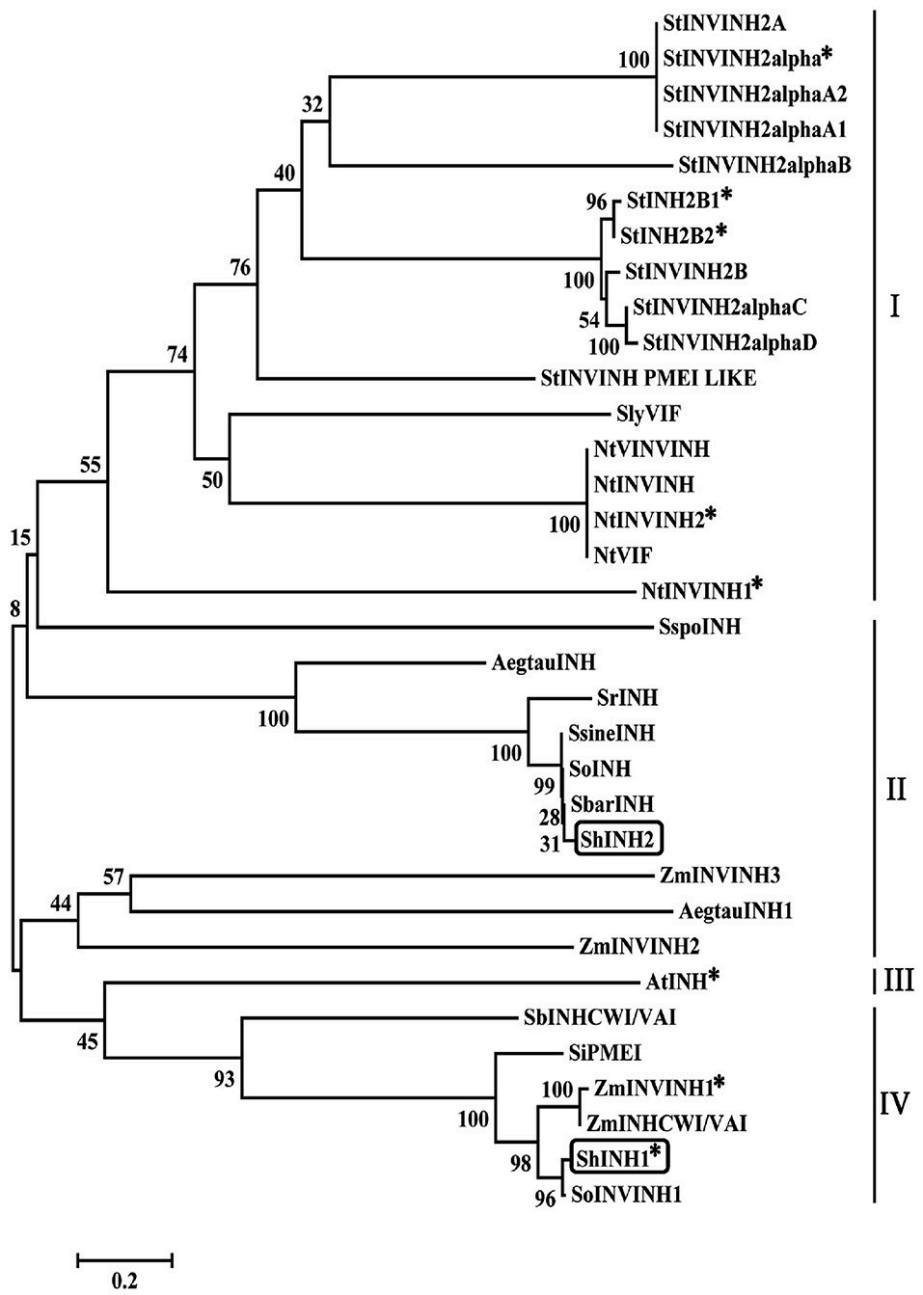
Phylogenetic relationships of plant INVINH proteins. The phylogenetic tree was constructed by neighbor joining using aligned INVINH sequences analyzed by MEGA7.0. Asterisk (^*^) indicates invertase inhibitors characterized experimentally. ShINH1 & ShINH2 are shown in boxes. Dicot (I and III) and monocot (II and IV) INVINH proteins alignment are shown in lines. INVINH-like proteins from *Solanum tuberosum* (StINVINH2A, GU321341; StINVINH2α, KJ788176; StINVINH2αA2, FJ810206; StINVINH2αA1, FJ810205; StINVINH2αB, FJ810207; StINVINH2b1, ADZ54776; StINVINH2b2, GU980595; StINVINH2B, GU321342; StINVINH2αC, FJ810208; StINVINH2αD, FJ810209; StINVINH PMEI LIKE,); *Solanum lycopersicum*, (KC007445); *Nicotiana tobacum* (NtVINVINH, AY145781; NtINVINH, AY594179; NtINVINH2, Y12805; NtVIF, AAN60076; NtINVINH1, Y12806); *S. spontaneum* (SspoINH, KP844455); *Aegilops tautschi* (AegtauINH, XM_020320985); *S. robustum* (SrINH, KP055631); *S. sinense* (SsineINH, KP997206); *S. officinarum* (SoINH, KP997207); *S. barberi* (SbarINH, KU057162); *Saccharum* hybrid (ShINH2, MG457817); *Zea mays* (ZmINVINH3, CAC69343); *A. tautschi* (AegtauINH1, XM_020311699); *Z. mays* (ZmINVINH2, CAC69336); *Arabidopsis thaliana* (AtINH, Y12807); *Sorghum bicolor* (SbINHCWI/VAI, XM_002446958.2); *Setaria indica* (SiPMEI, XM_004978185.1); *Z. mays* (ZmINVINH1; ZmINHCWI/VAI, XM_008670754.2) and *Saccharum* hybrid (ShINH1, MG457818; SoINVINH1, KF575171;). A total of 1,000 bootstrapping runs were performed and % reliability is labeled next to each branch. ShINH1 and ShINH2 clustered within the *Saccharum* spp. and were closely related to monocot INVINH proteins.

To elucidate the phylogenetic relationship between ShINH1, ShINH2 and known plant INVINH proteins, their amino acid sequences were aligned and a neighbor-joining tree constructed (Figure 2). ShINH1 and ShINH2 clustered together with monocot INVINH proteins. ShINH1 is most closely related to SoInvInh1, an uncharacterized INVINH of *Saccharum* hybrid, and closely related to *Z. mays* (ZmINVINH1 and ZmINHCWI/VAI), *Setaria indica* (SiPMEI) and *Sorghum bicolor* (SbINHCWI/VAI) INVINH proteins. ShINH2 aligned more closely with *Saccharum* spp. INVINH proteins (Figure 2).

### Localization of ShINH1

**Figure 3:**
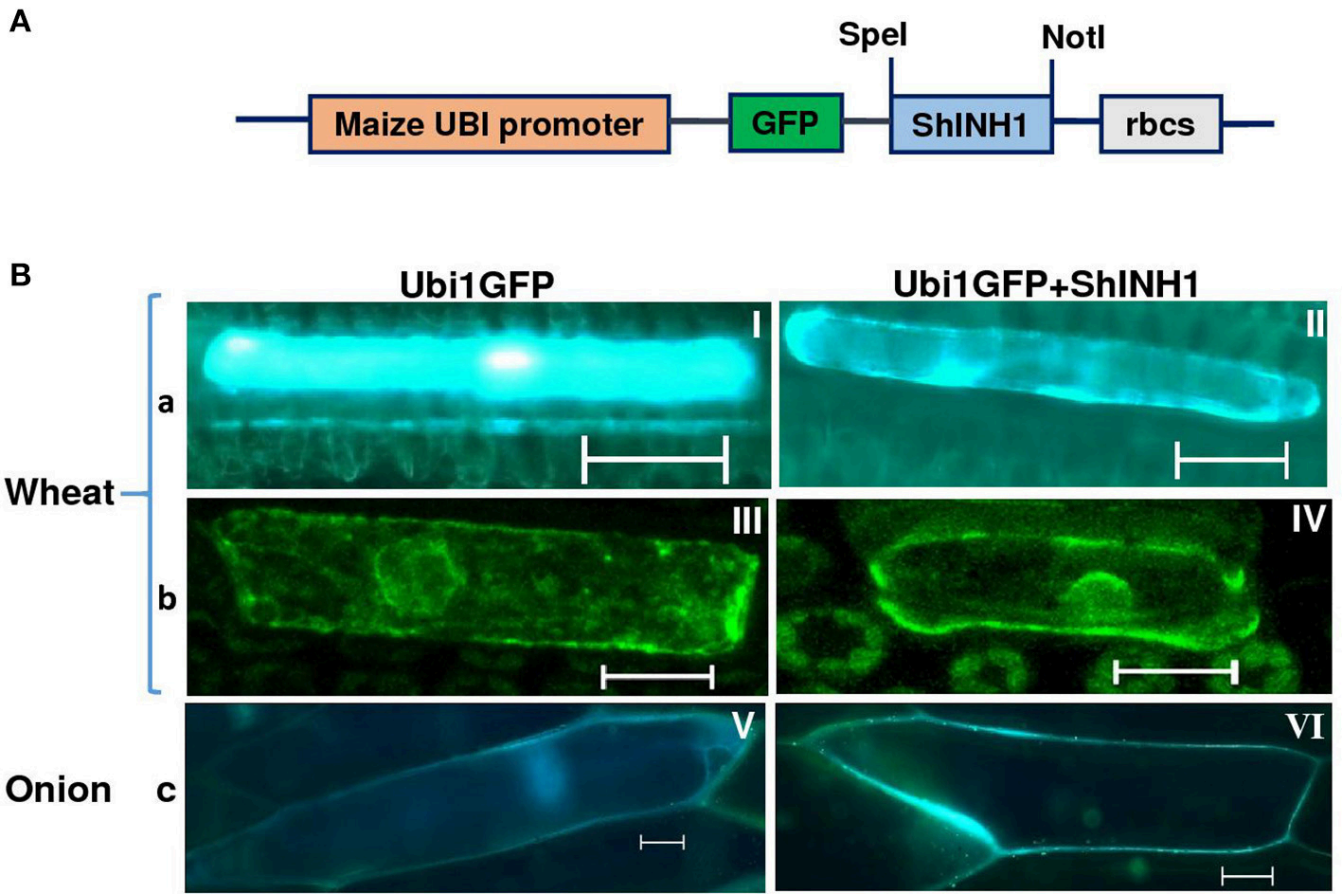
Subcellular localization of ShINH1 protein. **(A)** GFP-ShINH1 construct under control of the maize UBi1promoter. **(B)** Transient expression of UBi1GFP (control) and Ubi1GFP-ShINH1. Particle bombardment into wheat leaves and onion epidermal cells was performed and cells visualized using fluorescence and confocal microscopy 36 h after incubation at room temperature. (a and b) fluorescent and confocal images of wheat leaf cells. (c) Florescent image of onion epidermal cells. (I, III, and V: GFP controls) GFP accumulation in cytoplasm, nucleus and cell wall. (IV) Accumulation of GFP-ShINH1 in the cell wall, nucleus and cytoplasmic strands in wheat leaf cells. (II and VI) Enhanced fluorescence at the periphery of the cell indicates that GFP-ShINH1 localizes to the cell wall/apoplasmic region.

To examine the localization of ShINH1, we bombarded a GFP or ShINH1-GFP fusion construct into young wheat leaves and onion epidermal cells; in both constructs, gene expression is under control of the constitutive maize ubiquitin promoter (ZmUbi1; Figure 3A). In GFP controls, fluorescence was observed throughout the cell, including the nucleus (Figure 3B, panels I, III and V). In contrast, GFP-ShINH1 had a distinct localization pattern. No fluorescence signals were detected in the vacuole, and fluorescence in the nucleus and cytoplasm was reduced, whereas intense GFP fluorescence was detected at the periphery of wheat and onion cells (Figure 3B, panels II and VI), consistent with secretion of GFP-ShINH1 to the apoplast. The small amount of fluorescent signal in the cytoplasm might be due to the presence of transitory GFP-ShINH1 in the secretory pathway to the cell wall, (Figure 3B, panel II), which could be a feature of apoplastic proteins. Our data suggest that ShINH1 is predominantly localized to the apoplast and is a cell wall INVINH. However, confocal microscopic observation of wheat cells detected strong fluorescence signals in the cell wall and nucleus (Figure 3B, panel IV), in contrast to the results of fluorescence microscopic observation. Further detailed study is required to understand and validate the nature of ShINH1 localization in sugarcane.

### Temporal and spatial expression of *ShINH1*

**Figure 4:**
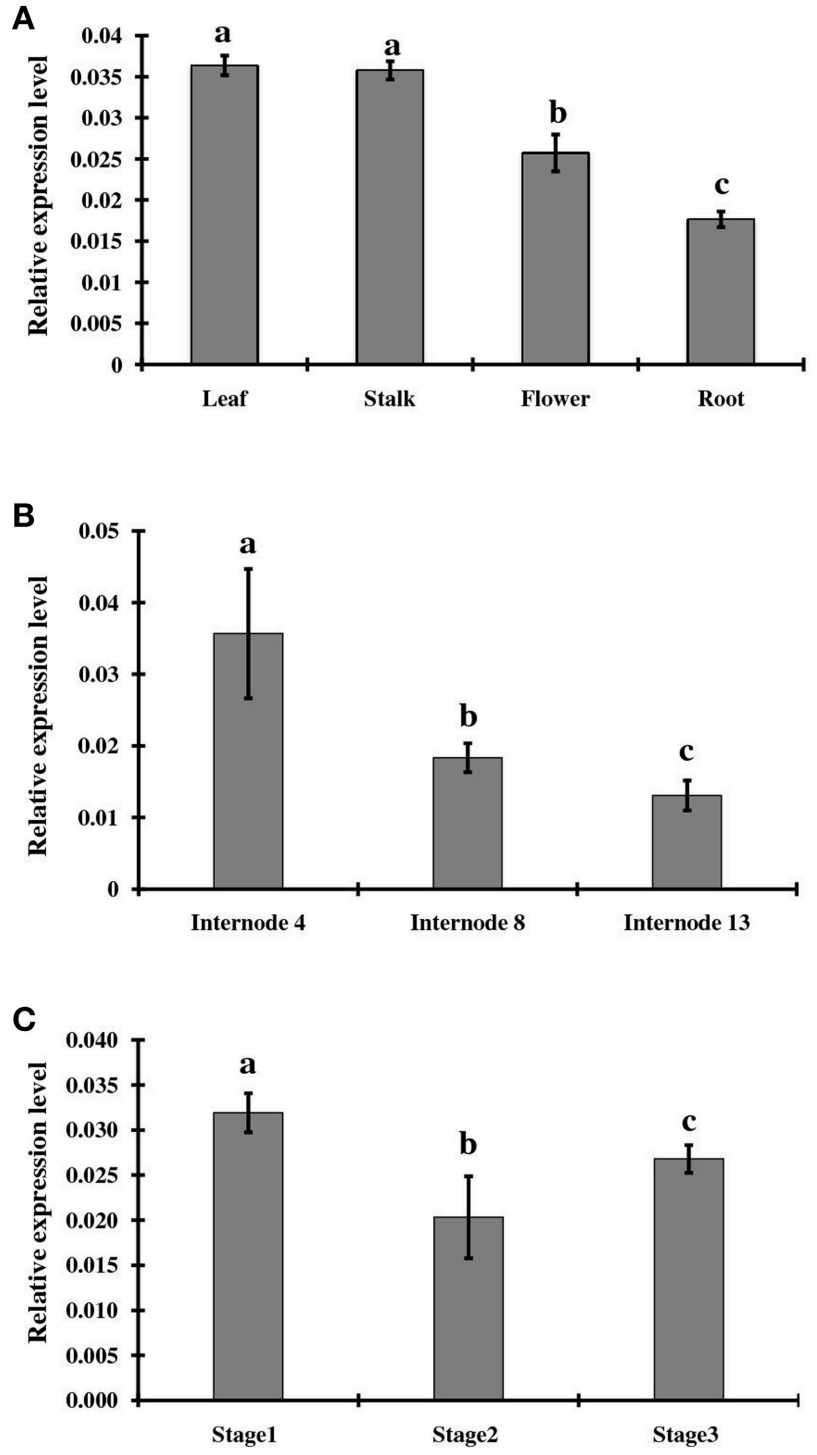
Relative expression levels of *ShINH1* in sugarcane. **(A)** Tissue specific expression of *ShINH1* in leaf, stalk, flower, and root tissues obtained from mature sugarcane plants. Significantly increased expression of *ShINH1* was observed in leaf and stalk as compared to flower and root. **(B)** Developmental expression of *ShINH1* in sugarcane stem during sucrose accumulation. Analysis of internodes I4 (young), I8 (moderately mature) and I13 (fully mature) from pooled internodal tissues revealed significant expression of *ShINH1* in I4 compared to I8 and I13. **(C)** Relative expression levels of *ShINH1* during flower development. Flowers from stage 1 (young flowers), stage 2 (moderately mature) and stage 3 (fully mature flowers) were used. Values are mean ± *SD* of 3 biological replicates. Lower case letters with same or different alphabets indicate statistically non-significant and significant respectively (Duncan's multiple range test; *P* < 0.01).

To examine tissue-specific and developmental expression of *ShINH1*, we extracted RNA from various sugarcane tissues, including leaf, root, flower, and stalk, and measured expression levels using qRT-PCR (Figure 4A). S*hINH1* transcript levels were significantly lower in root and flower compared to leaf and stalk. Expression was lowest in roots as compared to leaf (2.1-fold higher), stalk (2.05-fold higher), and flowers (1.4-fold higher) (Figure 4A). We also analyzed the expression of *ShINH1* at different stages of stalk development by quantifying transcript levels in internodal tissues I4, I8 and I13. *ShINH1* expression was significantly higher (1.94- and 2.69-fold) in young immature internodes (I4) compared with moderately (I8) and fully (I13) mature internodes (Figure 4B). The decrease in *ShINH1* expression during stalk maturation may suggest a regulatory role for this INVINH in sucrose accumulation during stalk development.

We also studied *ShINH1* expression in flowers at different stages of maturity. Expression of *ShINH1* in young flowers (Stage 1) was 1.5- and 1.2-fold higher, respectively, than in moderately mature (Stage 2) and fully mature (Stage 3) flowers (Figure 4C). Although there are significant differences in *ShINH1* expression between flowering stages, there was no clear correlation between gene expression level and flower maturity.

### Characterization of recombinant ShINH1

**Figure 5:**
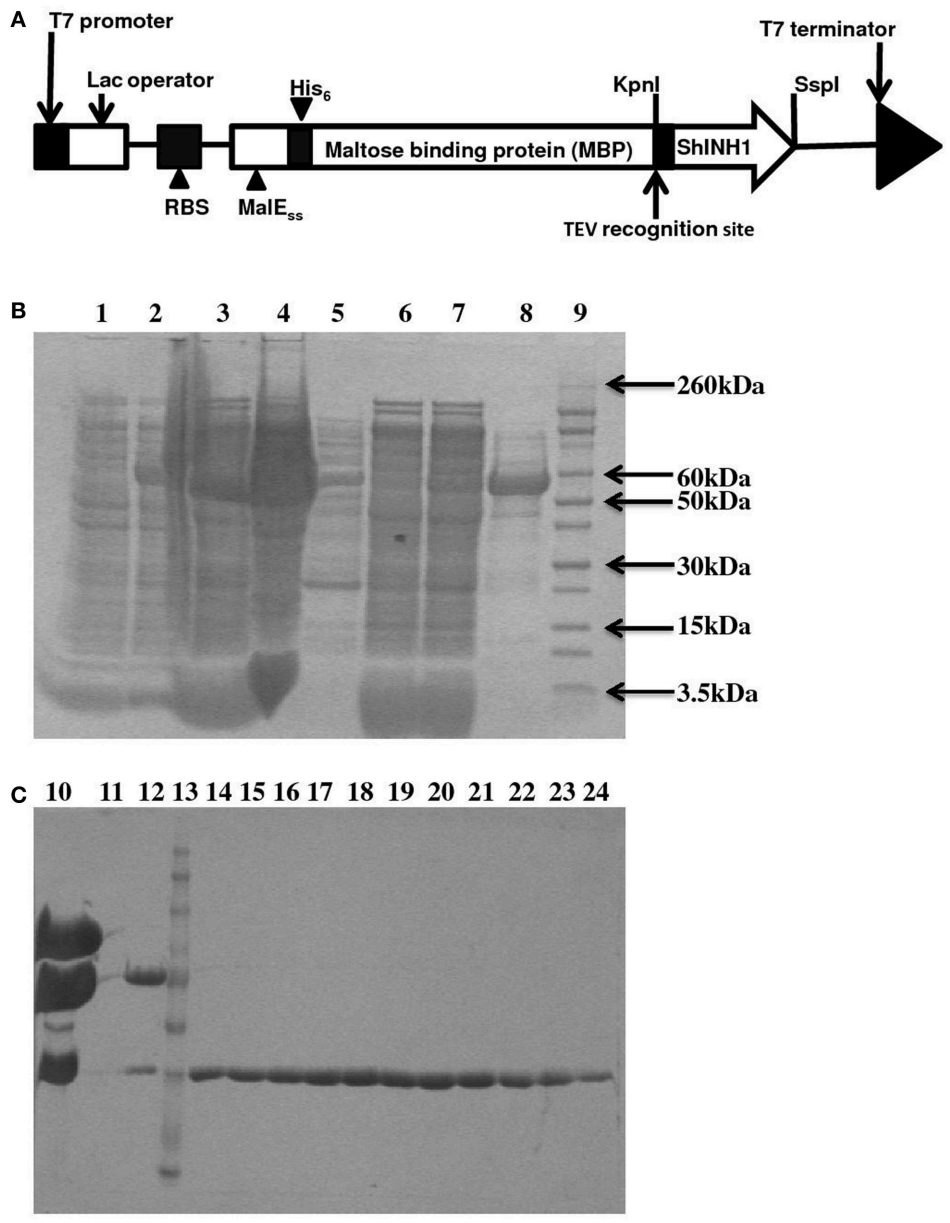
Recombinant ShINH1 expression in *E. coli*. **(A)** Schematic of construct encoding a His_6_-MBP: ShINH1 fusion protein for production of recombinant ShINH1 in *E. coli*. SDS-PAGE gels showing expression and purification of recombinant ShINH1: **(B)** Production and NiNTA purification of recombinant ShINH1 and **(C)** TEV cleavage and purification of recombinant ShINH1 through gel filtration column chromatography. Lane1: Pre induction sample; Lane2: IPTG induction sample; Lane3: Insoluble fraction; Lane4: Soluble fraction; Lane5: flow through; Lane6: 10 mM imidazole wash; Lane7: 30 mM Imidazole wash; Lane8: 300 mM imidazole wash; Lane9: Molecular size marker (Novex Sharp pre-stained protein ladder; Thermofisher Scientific, USA); Lane10: TEV protease treated sample; Lane12: Fraction containing cleaved His_6_-MBP and ShINH1; Lane13: Molecular size marker; Lanes14–24: Purified ShINH1 Samples.

**Figure 6:**
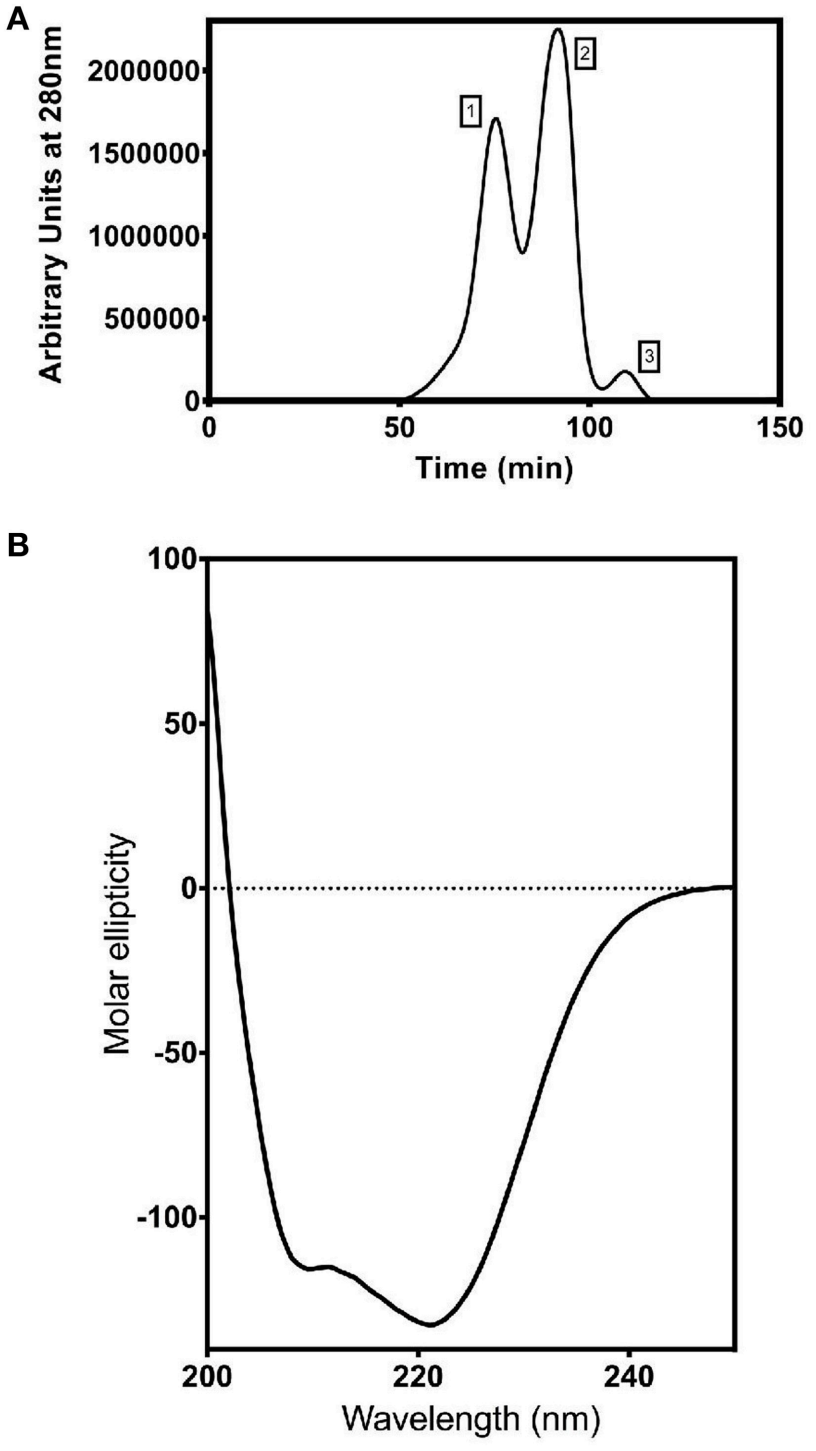
Purification and characterization of recombinant ShINH1. **(A)** Fractionation of recombinant ShINH1 using gel filtration chromatography. A HiTrap S-200 prepacked gel filtration column was used to separate TEV cleaved recombinant ShINH1 (peak 3) from uncleaved (His_6_-MBP: ShINH1 as peak 1) and His_6_-MBP (peak 2) fractions shown in the chromatogram. **(B)** Circular dichroism (CD) spectrum of recombinant ShINH1 obtained at 25°C.The CD spectrum contains minima at 222 and 209 nm, which are diagnostic of α-helical secondary structure.

To determine whether ShINH1 functions as an invertase inhibitor *in vitro*, we expressed recombinant ShINH1 in *E. coli* and purified the recombinant protein to homogeneity using a combination of nickel affinity and gel filtration chromatography (Figures 5B, C, 6A). The *E. coli* periplasmic expression system yielded ~1.0 mg/L of soluble ShINH1. The CD spectrum of recombinant ShINH1 contained minima at 222 and 209 nm, which are diagnostic of α-helical secondary structure (Figure 6B). The CD spectrum is consistent with the secondary structure predicted by PSIPRED (Figure S8) and the structurally well-characterized tobacco invertase inhibitor (Hothorn et al., 2010).

**Figure 7:**
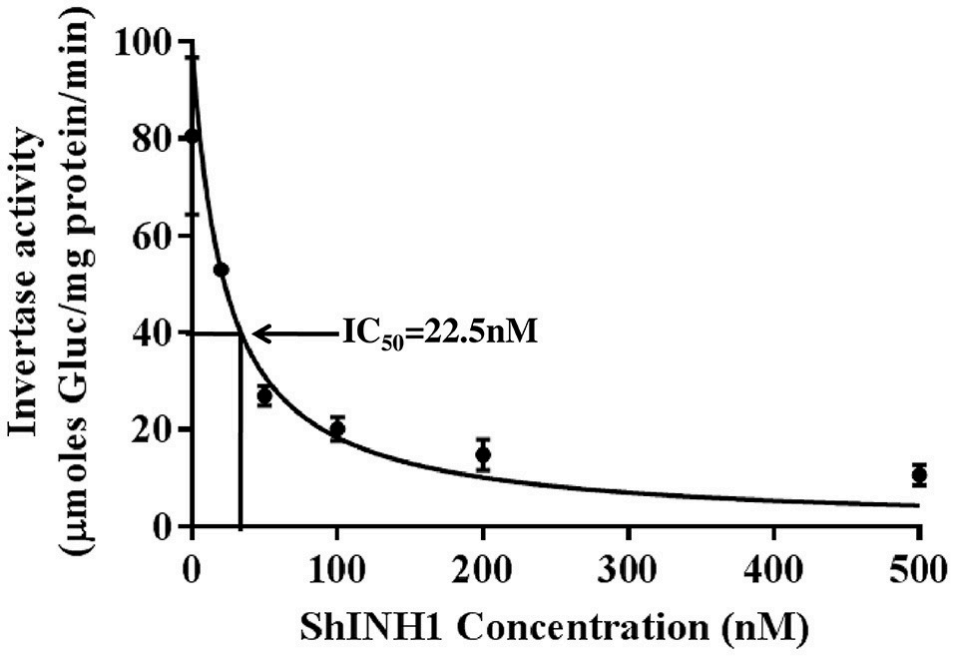
Recombinant ShINH1 inhibits invertase *in vitro*. The activity of commercial acid invertase (Sigma) was measured after pre-incubation with 0–500 nM recombinant ShINH1 and the concentration of ShINH1 that yields 50% inhibition calculated as the IC_50_ using GraphPad Prism 7.04 software. Data are presented as mean ± *SD* of three reactions. Statistical significance was compared between the different ShINH1 concentrations (Duncan's multiple range test; *P* < 0.01).

Recombinant ShINH1 was found to be a potent inhibitor of acid invertase, with the concentration-response data yielding an IC_50_ of 22.5 nM (Figure 7). This potent inhibitory activity is consistent with ShINH1 playing a role in post-translational regulation of sugarcane invertases.

## Discussion

In the present study, we identified two INVINH gene homologs (*ShINH1* and *ShINH2*) in sugarcane and, for the first time, experimentally demonstrated that sugarcane INVINH proteins are potent invertase inhibitors.

INVINH genes have been discovered in numerous plant species (Greiner et al., 1999; Bate et al., 2004; Brummell et al., 2011; Qin et al., 2016). Amongst the *Saccharum* spp., six uncharacterized INVINH genes, in addition to ShINH1 and ShINH2, have been reported (Figure 1 and Figure S7). The features of the ShINH1 and ShINH2 proteins clearly place them within the family of invertase inhibitors. Both proteins possess four cysteine residues, predicted to form two disulfide bridges, a conserved feature across all plant INVINH proteins (Figure 1; Bate et al., 2004; Rausch and Greiner, 2004; Brummell et al., 2011), and CD analysis of ShINH1 revealed a high proportion of α-helical secondary structure, consistent with the tobacco homolog (Hothorn et al., 2004; Rausch and Greiner, 2004). Phylogenetic analysis revealed that ShINH1 and ShINH2 are most closely related to other monocot INVINH sequences (Figure 2).

Many plant species appear to contain multiple INVINH isoforms that are directed to separate subcellular compartments by protein targeting elements. For example, in tobacco, NtINH is vacuolar while the related NtINH1 is located in the cell wall (Weil et al., 1994; Greiner et al., 1998). Similarly, in potato, INH1 is localized to the cytoplasm and cell wall, whereas INH2 is found in the cytoplasm and vacuole (Brummell et al., 2011). The putative signal peptides in ShINH1 and ShINH2 are predicted to direct the mature proteins to the endoplasmic reticulum for ultimate secretion to the apoplast and vacuole, respectively (Figures S5, S6). This is consistent with our observation that ShINH1-GFP fusion protein was detected primarily in the cell wall in both wheat leaves and onion epidermal cells (Figure 3), although this needs further investigation, as some cellular signal was also observed. Although localization of ShINH2 was not experimentally examined, we predict it is localized to the vacuole.

In general, tissue specific expression of INVINH genes varies among plant species depending on the source-sink dynamics. As INVINH proteins are integral modulators of invertase, they are frequently expressed in similar developmental and anatomical patterns to invertase enzymes. For example, it has been proposed that kernel-specific expression of ZmINH1 has a regulatory role in endosperm and embryo development in maize, helping to maintain hexose/sucrose ratios by controlling the activity of apoplasmic invertase (Bate et al., 2004). In potato, where invertase and its inhibitors have been extensively characterized, starch is the major carbohydrate accumulated in the tubers, which act as sinks for photosynthate. During tuber growth, reduced expression of potato INVINH genes favors starch accumulation over sucrose because the invertase is an essential step in conversion of sucrose to starch precursors. However, cold-induced sweetening of potato tubers may occur through the activity of invertase during post-harvest and this was successfully reduced by high expression of StINH mRNA in tubers (Greiner et al., 1999; Brummell et al., 2011).

In sugarcane, *ShINH1* is more strongly expressed in leaf and stalk than root and flowers (Figure 4A), although some expression was seen throughout the plant. The current study also revealed that *ShINH1* expression is developmentally regulated during internodal maturation, with higher levels in young internodes (I4) compared to moderately mature (I8) and fully mature internode (I13) (Figure 4B). It has been reported that expression of invertase enzymes is higher in young sugarcane tissues where rapid sucrose hydrolysis occurs to provide substrates for cell wall biosynthesis and other reactions (Zhu et al., 1997; Chandra et al., 2015). It is likely that higher expression of *ShINH1* in young internodes is part of a post-translational control mechanism involving feedback regulation of sucrose biosynthesis and degradation where invertase enzymes exist. Similarly in the storage tissues of mature internodes, ShINH1 may add an additional mechanism to control futile cycling between sucrose and hexoses to regulate sucrose accumulation in the stalk.

We examined expression of ShINH1 in inflorescences to elucidate its role in carbon partitioning during development of reproductive structures, based on the role of the INH1 homolog in other species. It was previously shown that expression of StINH1 in potato (Brummell et al., 2011), NtINH1 in tobacco (Greiner et al., 1998) and SolyCIF in tomato (Reca et al., 2008) is highest in flowers. In tomato, a vacuolar-targeted INVINH plays an important role in sucrose-mediated fruit ripening by regulating the activity of a ripening inhibitor (Qin et al., 2016). Although *ShINH1* was expressed in sugarcane flowers at three developmental stages, including early seed development (Figure 4C), this was not the tissue with the highest expression level and there was no clear correlation with maturation. This may reflect the lack of fruit structures in monocot seeds together with the small size of sugarcane seeds and their relatively low starch content (Siqueira et al., 2015).

Considering the complexity of sucrose metabolism in sugarcane due to its multiple genes and intricate source-sink dynamics, it will be essential to understand the molecular regulation of sucrose accumulation in order to improve yields (Rae et al., 2005; Chandra et al., 2011; Suresha et al., 2017). There are still many unanswered questions about the capacity of sugarcane sink tissue to store sucrose and its requirement and mechanism of partitioning for growth and development of the plant. Previous approaches to understanding sink strength and transport kinetics have used transcriptomic analyses to identify key genes and pathways (Casu et al., 2005; Watt et al., 2005). Post-translational regulation has received less attention, partly because it has been difficult to address in this non-model system lacking the resources of complete genome and metabolome profiles. The recent availability of sugarcane genome information has now allowed us to identify a regulator of enzyme activity which could open new avenues to manipulation of sucrose accumulation.

Ectopic expression of INVINH proteins from tobacco (Greiner et al., 1999) and potato (Brummell et al., 2011) in transgenic potato tubers has been used to control invertase activity. A similar approach may be effective in sugarcane, assisted by the availability of well-established transformation systems and protocols for subcellular targeting of proteins for *in planta* expression (Jackson et al., 2007, 2010; Rae et al., 2009, 2010; Palaniswamy et al., 2016). Furthermore, by using inducible expression, it may be possible to increase INVINH expression specifically at the time of harvest so as to reduce post-harvest loss of sucrose. Chandra et al. (2014) reported that acid invertase enzymes are involved in the degradation of sucrose during cane maturity and post-harvest. In addition to endogenous invertases, micro-organisms that colonize and grow in cut sugarcane stalks also produce invertases that further contribute to sucrose loss. Application of anti-microbial formulations at the time of harvest slows the rate of sucrose breakdown but losses are still substantial (Singh et al., 2008). It is likely that plant invertase inhibitors would be effective against both plant and microbial invertases. The advantage of functional specificity of ShINH1 established in the present work against soluble acid invertase provides a new avenue that may pave the way for controlling the activity of invertase enzymes during growth and prevent sucrose losses during the post-harvest period. In addition to controlling sucrose accumulation, it has been shown that invertases play important roles in regulation of various physiological processes like seed development and maturity, leaf senescence, drought and cold stresses and defense pathways (Jin et al., 2009; Bonfig et al., 2010; Ni, 2012; Chen et al., 2016; Tang et al., 2017; Xu et al., 2017; Zuma et al., 2018). Manipulation of invertases through specific INVINHs may lead to physiological and biochemical changes in plants through control of metabolic flux and source-sink balance of sugars (Roitsch and Gonzalez, 2004; Tauzin and Giardina, 2014). Therefore, INVINHs are valuable candidates for post-translational regulation of invertases through which many traits of interest can be modified to enhance crop yield.

## Conclusions

In this study, we identified two sugarcane invertase inhibitors (*ShINH1* and *ShINH2*) and demonstrated the function of ShINH1 against an invertase enzyme. This is the first report to ascertain the functional identity of a sugarcane invertase inhibitor. We also provided the evidence for a role for ShINH1 in regulation of sucrose accumulation by studying its temporal and spatial expression in sugarcane. The functional significance of sugarcane invertase inhibitors established in this study offers new opportunities to enhance the yield of sucrose and sugar recovery by regulating the activity of invertase enzymes and preventing pre-and post-harvest sucrose deterioration, although careful attention would be required to monitor changes in the growth and development of transgenic plants. Nevertheless, precise targeting of vacuolar invertases by using stalk specific wound/harvest inducible promoters has the potential to have more impact on sucrose yield which is yet to be accomplished in sugarcane.

## Accession numbers

The nucleotide sequences for *ShINH1* and *ShINH2* reported in this manuscript have been submitted to GenBank with accession numbers MG457817 and MG457818, respectively.

## Author contributions

SS: conceived, designed, and performed the experiments, analyzed data, and drafted the manuscript; RA: contributed to protein expression, purification, enzyme assays, CD analyses, and manuscript preparation; SK: contributed to gene expression analysis and GFP localization experiments; DG: contributed to generation of sugarcane tissue samples and genes cloning; GK: advised on protein expression and contributed to manuscript writing; AR: contributed to collection of flower samples, manuscript preparation and editing, and supervised all experiments.

### Conflict of interest statement

The authors declare that the research was conducted in the absence of any commercial or financial relationships that could be construed as a potential conflict of interest. The reviewer JN and handling Editor declared their shared affiliation.

## Supplementary material

Figure S1 Sugarcane flowers of different maturity stages used for *ShINH1* expression analysis. Flowers were collected from field-grown plants based on level of maturity. Stage 1 (young flowers), stage2 (moderately mature), and stage3 (fully mature) in three replicates are shown. Both top and bottom parts of each were used for expression analysis of *ShINH1*.

Figure S2 *Zea mays* (*ZmINH*; NM_001157609) and *Nicotiana tabacum* (*NtINH*; Y12806) INVINH sequences were used to search for potential sugarcane INVINH sequences via a BlastN search against the sugarcane genome database. Three hits were obtained using the *ZmINH* sequence, but none were obtained using the *NtINH* sequence.

Figure S3 Isolation of sugarcane INVINH genes (*ShINH1* and *ShINH2*). PCR amplification of stem cDNA using gene-specific primers and *Eco*RI-digested pGEM-T–*ShINH1*/*INH2* recombinant plasmids are shown using1%agarose gels. Lane M: 1 kb DNA ladder; Lane 1:ShINH1 PCR-amplified product; Lanes 2–7: *Eco*RI-restricted ShINH1 recombinant plasmids; Lanes 9–10: ShINH2 PCR amplified product; Lanes 11–16: *Eco*RI-restricted ShINH2 recombinant plasmids.

Figure S4 Nucleotide and deduced amino acid sequences of the *ShINH1* and *ShINH2*genes. **(A)** Alignment of nucleotide sequences consisting of 531 and 576 bp ORFs with start codon ATG and stop codon TAG and TAA respectively. **(B)** Alignment of deduced amino acid sequences of ShINH1 and ShINH2 genes consisting of 177 and 192 amino acid residues respectively. Putative signal peptides are marked with a box in both the nucleotide and amino acid sequences.

Figure S5 Prediction of signal peptide in the ShINH1 & ShINH2 proteins. The deduced amino acid sequences of ShINH1 and ShINH2 were analyzed for signal peptide sequences using Predisi (www.predisi.de). A signal peptide was identified with high confidence (Predisi score of 0.87 on a 0–1 scoring scale) at **(A)** residues 1–21 of ShINH1 and **(B)** residues 1–19 of ShINH2.

Figure S6 Prediction of subcellular localization of ShINH1 & ShINH2. Analysis of the deduced amino acid sequences of ShINH1 and ShINH2 proteins were analyzed for subcellular localization signals using PSORT (https://wolfpsort.hgc.jp) revealed that **(A)** ShINH1 is likely targeted to an extracellular location (i.e., cellwall/apoplasmic space; indicated in green), whereas **(B)** ShINH2 is predicted to be localized in the vacuolar lumen (indicated in red). Names of identical protein hits, localization, distance score, % identity and localization prediction are shown with lower distance scores indicating higher prediction values. Localization sites are indicated as extr, extracellular; chlo, chloroplast; nucl, nucleus; mito, mitochondria; cyto, cytoplasm; vacu, vacuole.

Figure S7 Pairwise comparison of deduced amino acid sequences of ShINH1 & ShINH2 with plant INVINH-like proteins from *S. sinense* (SsineINH, KP997206); *Saccharum* hybrid (ShINH2); *S. officinarum* (SoINH, KP997207); *S. barberi* (SbarINH, KU057162); *S. spontaneum* (SspoINH, KP844455); *S. robustum* (SrINH, KP055631), *Zea mays* (ZmIN VINH, EU969422); *Aegilops tautschi* (AegtauINH, XM_020320985; AegtauINH1, XM_020311699); *Saccharum* hybrid (SoINVInh1, KF575171; ShINH1); *Sorghum bicolor* (SbINH, XM_002453079; SbINH1, XM_002453080; SbINH2, XM_002452686 SbINHCWI/VAI, XM_002446958.2), *Zea mays* (INVINH1, EU952678; INVINH2; EU960562; ZmINHCWI/VAI, XM_008670754.2); *Setaria indica* (SiPMEI, XM_004978185.1); *Arabidopsis thaliana* (AtINH, Y12807; AtINVINH1, DQ056716); *Solanum tuberosum* (StINVINH2α, StINVINH2α, KJ788176; StINVINH2αB, FJ810207; StINVINH2αC, FJ810208; StINVINH2αD, FJ810209; StINVINH, JQ269669; StINVINH2B, GU321342), and *Nicotiana tabacum* (NtVINVINH, AY145781; NtINVINH, AY594179; NtINVINH2, Y12805; NtINVINH1, Y12806). Values indicate % similarity of INVINH proteins against each other. Colored boxes with numbers indicate degree of similarity: Light to dark red indicates moderate to high similarity whereas light to dark blue indicates moderately low to least similarity.

Figure S8 Prediction of secondary structure of ShINH1 and ShINH2 proteins. Deduced amino acid sequences of ShINH1 and ShINH2 proteins were analyzed for secondary structure using PSIPRED tool (http://bioinf.cs.ucl.ac.uk/psipred/). Both **(A)** ShINH1 and **(B)** ShINH2 were predicted to form predominantly α-helical secondary structure. Helical structure is indicated by the letter “H” and pink cylinders whereas, coil regions are indicated by the letter “C” and horizontal lines. Confidence of prediction is shown by the blue colored bars.
