# Supplementary figures and images for: Identification and Functional Characterization of Sugarcane Invertase Inhibitor (ShINH1): A Potential Candidate for Reducing Pre- and Post-harvest Loss of Sucrose in Sugarcane
Source: Front Plant Sci. 2018 May 3;9:598. doi: 10.3389/fpls.2018.00598 (PMC5944049; doi:10.3389/fpls.2018.00598)

**Replication 1**

**Replication 2**

**Replication 3**

**Stage 1**

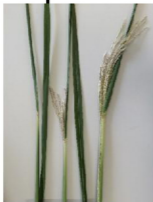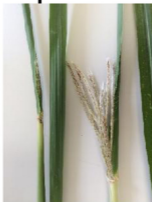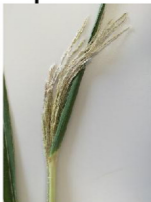

**Stage 2**

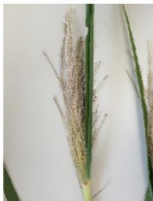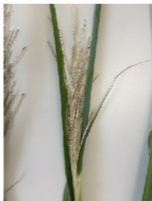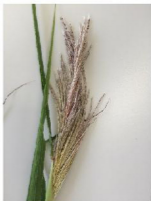

**Stage 3**

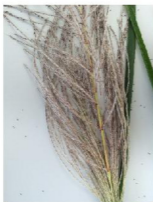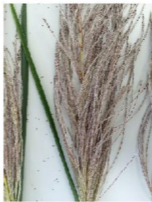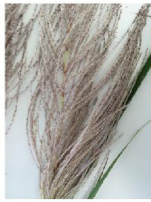

Supplement: Figure S1 — Sugarcane flowers of different maturity stages used for ShINH1 expression analysis. Flowers were collected from field-grown plants based on level of maturity. Stage 1 (young flowers), stage2 (moderately mature), and stage3 (fully mature) in three replicates are shown. Both top and bottom parts of each were used for expression analysis of ShINH1. [file Image_1.PDF]

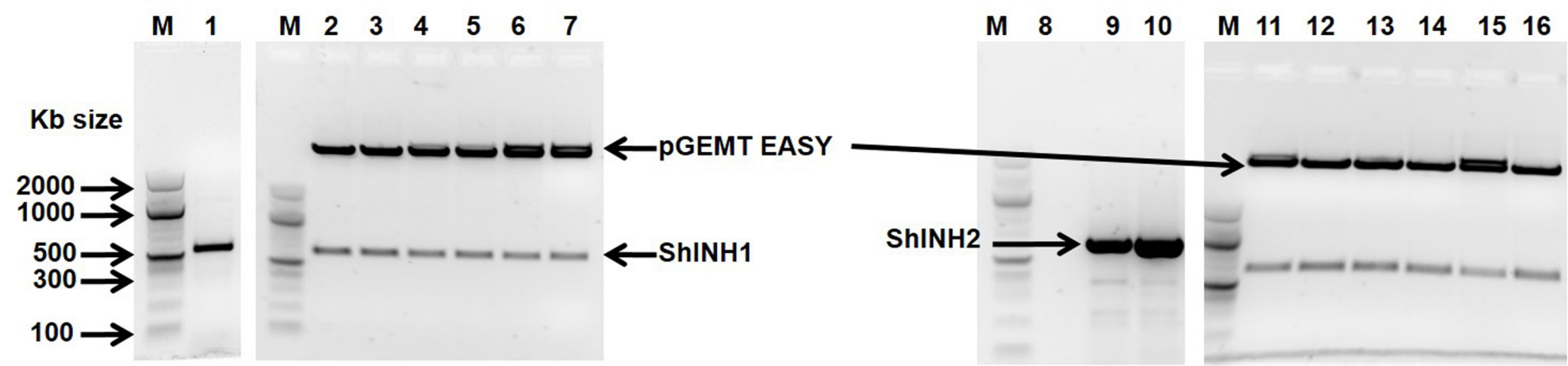

Supplement: Figure S3 — Isolation of sugarcane INVINH genes (ShINH1 and ShINH2). PCR amplification of stem cDNA using gene-specific primers and EcoRI-digested pGEM-T–ShINH1/INH2 recombinant plasmids are shown using1%agarose gels. Lane M: 1 kb DNA ladder; Lane 1:ShINH1 PCR-amplified product; Lanes 2–7: EcoRI-restricted ShINH1 recombinant plasmids; Lanes 9–10: ShINH2 PCR amplified product; Lanes 11–16: EcoRI-restricted ShINH2 recombinant plasmids. [file Image_3.PDF]

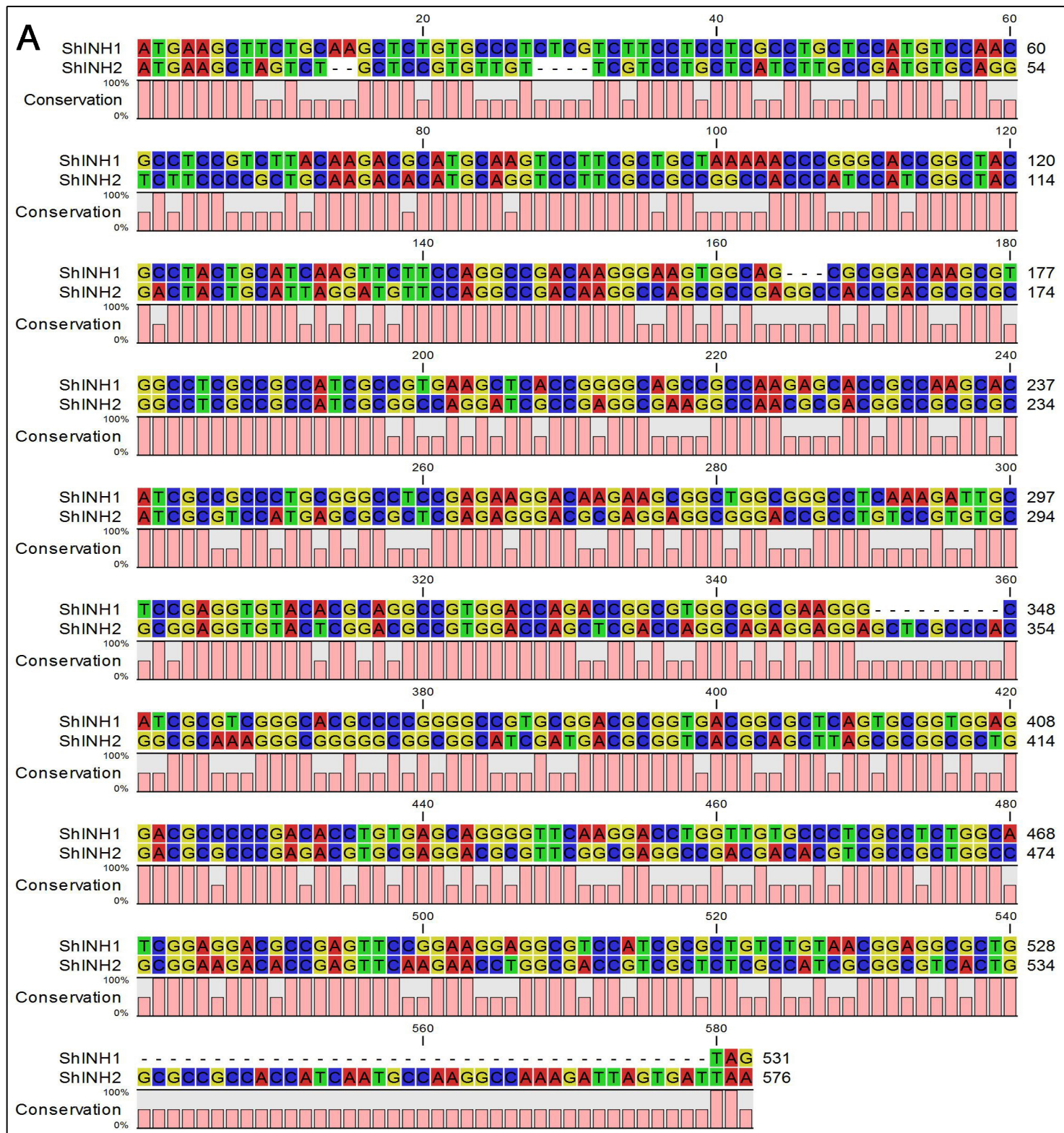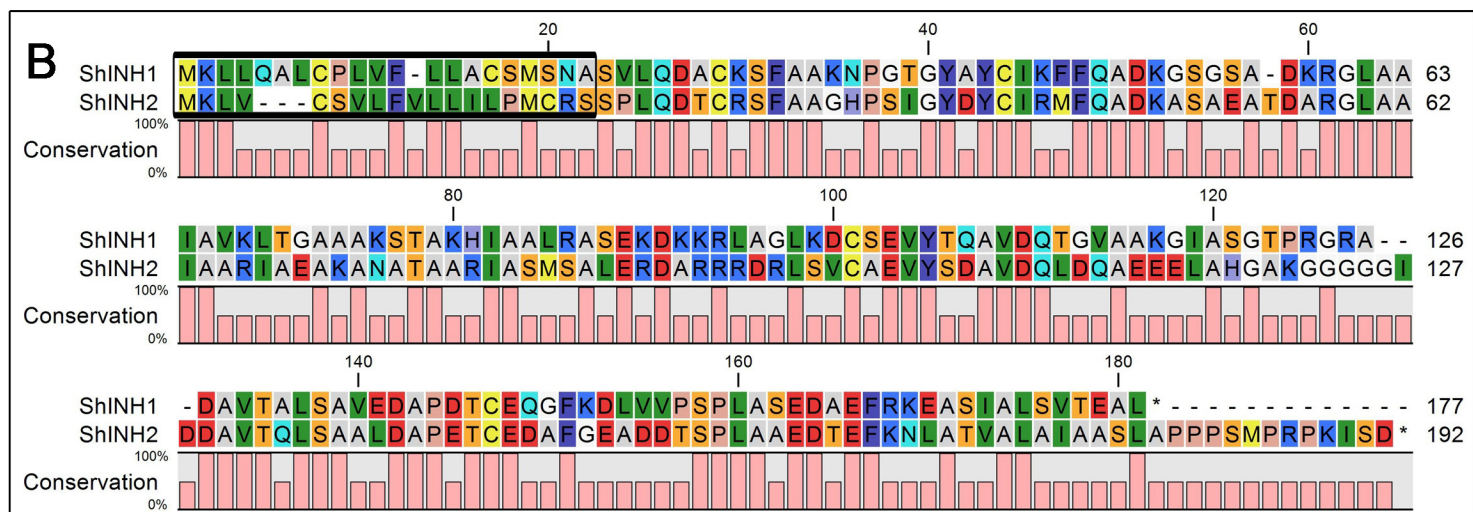

Supplement: Figure S4 — Nucleotide and deduced amino acid sequences of the ShINH1 and ShINH2genes. (A) Alignment of nucleotide sequences consisting of 531 and 576 bp ORFs with start codon ATG and stop codon TAG and TAA respectively. (B) Alignment of deduced amino acid sequences of ShINH1 and ShINH2 genes consisting of 177 and 192 amino acid residues respectively. Putative signal peptides are marked with a box in both the nucleotide and amino acid sequences. [file Image_4.PDF]

**A**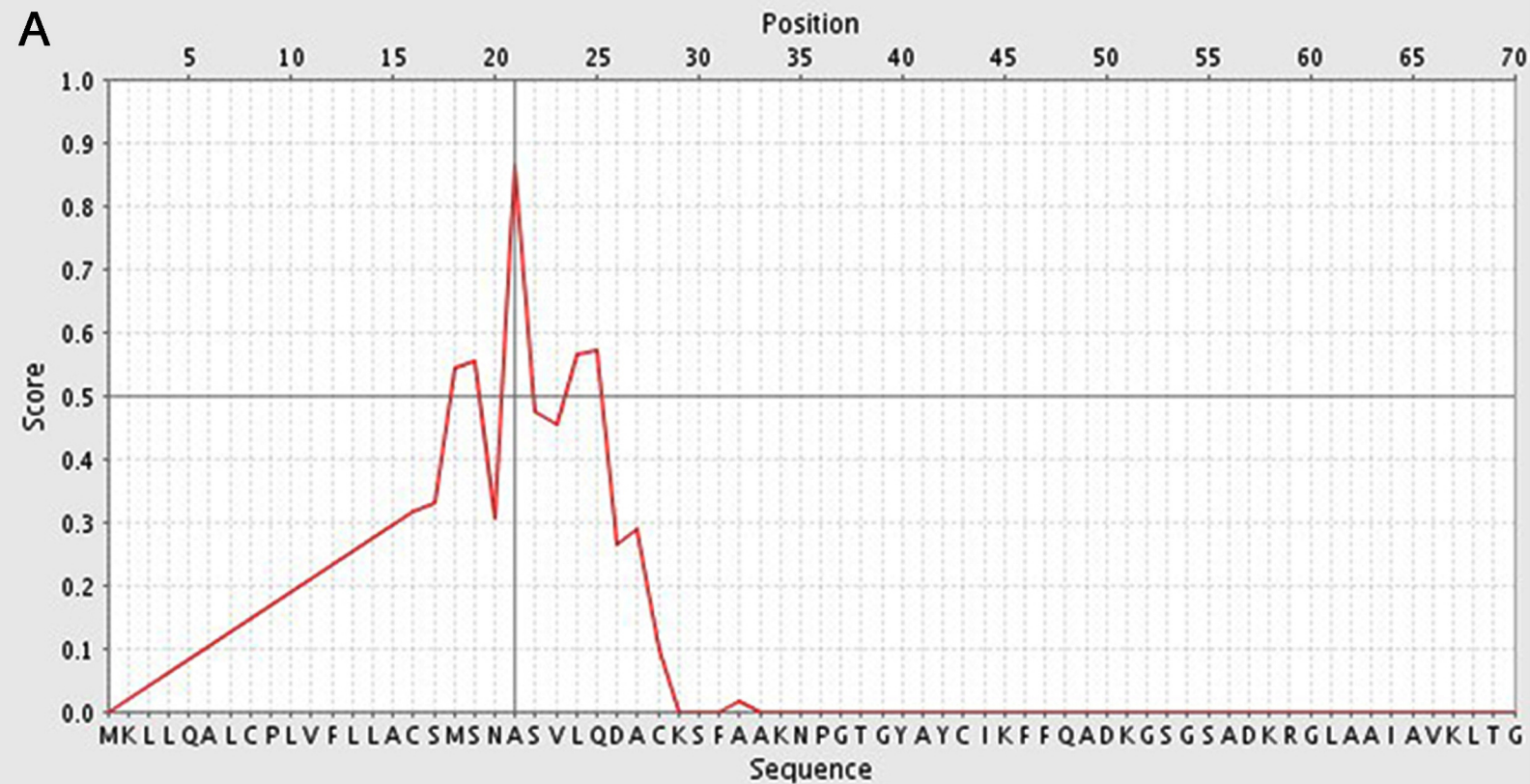**B**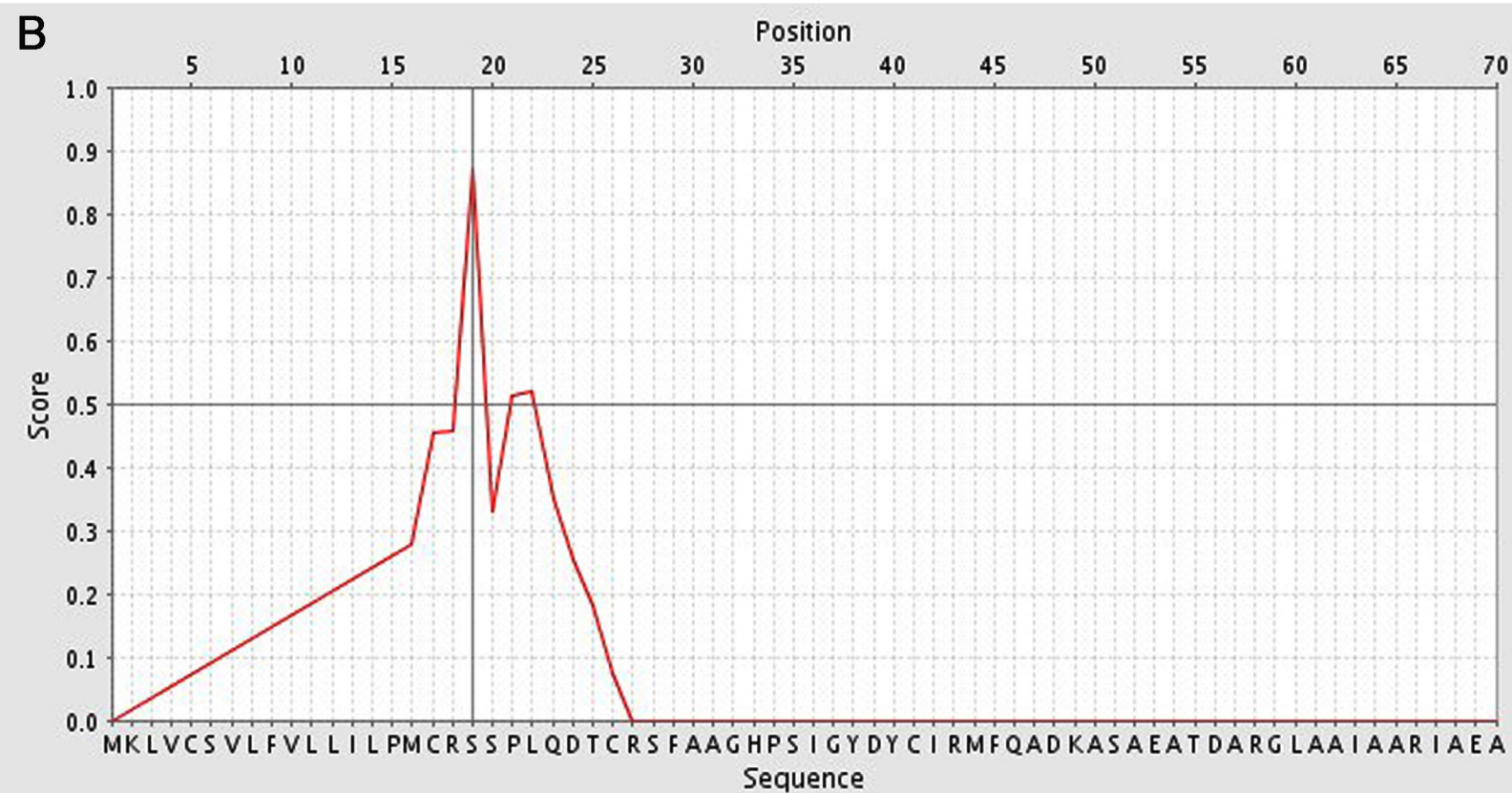

Supplement: Figure S5 — Prediction of signal peptide in the ShINH1 & ShINH2 proteins. The deduced amino acid sequences of ShINH1 and ShINH2 were analyzed for signal peptide sequences using Predisi (www.predisi.de). A signal peptide was identified with high confidence (Predisi score of 0.87 on a 0–1 scoring scale) at (A) residues 1–21 of ShINH1 and (B) residues 1–19 of ShINH2. [file Image_5.PDF]
