# Supplementary material for: Identification and Functional Characterization of Sugarcane Invertase Inhibitor (ShINH1): A Potential Candidate for Reducing Pre- and Post-harvest Loss of Sucrose in Sugarcane
Source: Front Plant Sci. 2018 May 3;9:598. doi: 10.3389/fpls.2018.00598 (PMC5944049; doi:10.3389/fpls.2018.00598)
Supplement: Figure S2 — Zea mays (ZmINH; NM_001157609) and Nicotiana tabacum (NtINH; Y12806) INVINH sequences were used to search for potential sugarcane INVINH sequences via a BlastN search against the sugarcane genome database. Three hits were obtained using the ZmINH sequence, but none were obtained using the NtINH sequence. [file Image_2.PDF]

```
# BLASTN 2.2.26+
# Query: gi|226507429|ref|NM_001157609.1| Zea mays invertase inhibitor (LOC100284714), mRNA
# Database: /srv/rocky2/Projects/Cultivar_sequencing/Sugarcane_R570/test_kanga/TestAssemblyPE/Scaffolded/R570_32kb_MP.Filtered.assemb.scaffold.errorless_gt1k
# Fields: query id, subject id, % identity, alignment length, mismatches, gap opens, q. start, q. end, s. start, s. end, evalue, bit score
# 3 hits found
gi|226507429|ref|NM_001157609.1|	Scaff413473	90.47	640	43	13	81	716	1270	645	0.0	832
gi|226507429|ref|NM_001157609.1|	Scaff294351	90.36	249	18	5	469	716	2634	2391	3e-86	326
gi|226507429|ref|NM_001157609.1|	Scaff762782	89.42	208	13	6	81	286	1569	1369	2e-64	254
# BLASTN 2.2.26+
# Query: gi|2765241|emb|Y12806.1| Nicotiana tabacum mRNA for invertase inhibitor homologue
# Database: /srv/rocky2/Projects/Cultivar_sequencing/Sugarcane_R570/test_kanga/TestAssemblyPE/Scaffolded/R570_32kb_MP.Filtered.assemb.scaffold.errorless_gt1k
# 0 hits found
# BLASTN 2.2.26+
# Query: gi|2765239|emb|Y12805.1| Nicotiana tabacum mRNA for invertase inhibitor
# Database: /srv/rocky2/Projects/Cultivar_sequencing/Sugarcane_R570/test_kanga/TestAssemblyPE/Scaffolded/R570_32kb_MP.Filtered.assemb.scaffold.errorless_gt1k
# 0 hits found
```
