# Supplementary material for: Identification and Functional Characterization of Sugarcane Invertase Inhibitor (ShINH1): A Potential Candidate for Reducing Pre- and Post-harvest Loss of Sucrose in Sugarcane
Source: Front Plant Sci. 2018 May 3;9:598. doi: 10.3389/fpls.2018.00598 (PMC5944049; doi:10.3389/fpls.2018.00598)
Supplement: Figure S6 — Prediction of subcellular localization of ShINH1 & ShINH2. Analysis of the deduced amino acid sequences of ShINH1 and ShINH2 proteins were analyzed for subcellular localization signals using PSORT (https://wolfpsort.hgc.jp) revealed that (A) ShINH1 is likely targeted to an extracellular location (i.e., cellwall/apoplasmic space; indicated in green), whereas (B) ShINH2 is predicted to be localized in the vacuolar lumen (indicated in red). Names of identical protein hits, localization, distance score, % identity and localization prediction are shown with lower distance scores indicating higher prediction values. Localization sites are indicated as extr, extracellular; chlo, chloroplast; nucl, nucleus; mito, mitochondria; cyto, cytoplasm; vacu, vacuole. [file Image_6.PDF]

A

id	site	distance	identity	comments
IPRA_SAGSA	extr	186.679	17.1271%	[Uniprot] SWISS-PROT45:Secreted.
RBS_PINTH	chlo	246.999	9.03955%	[Uniprot] SWISS-PROT45:Chloroplast.
IPRB_SAGSA	extr	248.695	16.0221%	[Uniprot] SWISS-PROT45:Secreted.
CHIA_CICAR	extr	251.655	13.9932%	[Uniprot] SWISS-PROT45:Extracellular.
CHIP_BETVU	extr	255.673	15.625%	[Uniprot] SWISS-PROT45:Extracellular.
FER_MARPO	chlo	259.967	11.3636%	[Uniprot] SWISS-PROT45:Chloroplast.
PORA_CUCSA	chlo	270.895	10.0503%	[Uniprot] SWISS-PROT45:Chloroplast.
ADT2_WHEAT	mito	281.812	12.9909%	[Uniprot] SWISS-PROT45:Integral mem protein
POR_DAUCA	chlo	289.588	10.5528%	[Uniprot] SWISS-PROT45:Chloroplast.
At3g20060.1	nucl	291.346	14.2857%	[Arath]
ALFC_CHLRE	chlo	299.896	12.5668%	[Uniprot] SWISS-PROT45:Chloroplast.
CHIA_PHAAN	extr	310.222	16.443%	[Uniprot] SWISS-PROT45:Extracellular.
POR_PEA	chlo	310.94	13.7845%	[Uniprot] SWISS-PROT45:Chloroplast.
At3g61990.1	cyto	313.912	13.7457%	[Arath]

B

id	site	distance	identity	comments
DEF_NICAL	vacu	222.761	11.9171%	[Uniprot] SWISS-PROT45:Vacuolar.
At5g53490.1	chlo	258.189	14.4068%	[Arath] Subclass:thylakoid
DEF1_CAPAN	extr	278.623	8.37696%	[Uniprot] SWISS-PROT45:Secreted.
CHI4_BRANA	extr	288.109	13.806%	[Uniprot] SWISS-PROT45:Extracellular.
NTPA_PEA	nucl	292.005	12.0879%	[Uniprot] SWISS-PROT45:Nuclear.
AFP1_BRANA	extr	301.914	9.42408%	[Uniprot] SWISS-PROT45:Secreted.
At4g35350.1	vacu	310.296	14.6479%	[Arath]
MPH1_HOLLA	extr	311.553	12.4528%	[Uniprot] SWISS-PROT45:Secreted.
AFP3_BRANA	extr	317.083	10.4712%	[Uniprot] SWISS-PROT45:Secreted.
AMP1_MACIN	extr	317.598	15.1832%	[Uniprot] SWISS-PROT45:Secreted.
GAS4_ARATH	extr	318.556	8.90052%	[Uniprot] SWISS-PROT45:Secreted.
MDHM_CHLRE	mito	319.179	15.2815%	[Uniprot] SWISS-PROT45:Mitochondrial matrix.
FBH1_CUPWR	chlo	321.84	12.5%	[Uniprot] SWISS-PROT45:Chloroplast.
ASPR_CUCPE	vacu	332.85	9.74659%	[Uniprot] SWISS-PROT45:Vacuolar.
