# Supplementary material for: Identification and Functional Characterization of Sugarcane Invertase Inhibitor (ShINH1): A Potential Candidate for Reducing Pre- and Post-harvest Loss of Sucrose in Sugarcane
Source: Front Plant Sci. 2018 May 3;9:598. doi: 10.3389/fpls.2018.00598 (PMC5944049; doi:10.3389/fpls.2018.00598)
Supplement: Figure S7 — Pairwise comparison of deduced amino acid sequences of ShINH1 & ShINH2 with plant INVINH-like proteins from S. sinense (SsineINH, KP997206); Saccharum hybrid (ShINH2); S. officinarum (SoINH, KP997207); S. barberi (SbarINH, KU057162); S. spontaneum (SspoINH, KP844455); S. robustum (SrINH, KP055631), Zea mays (ZmIN VINH, EU969422); Aegilops tautschi (AegtauINH, XM_020320985; AegtauINH1, XM_020311699); Saccharum hybrid (SoINVInh1, KF575171; ShINH1); Sorghum bicolor (SbINH, XM_002453079; SbINH1, XM_002453080; SbINH2, XM_002452686 SbINHCWI/VAI, XM_002446958.2), Zea mays (INVINH1, EU952678; INVINH2; EU960562; ZmINHCWI/VAI, XM_008670754.2); Setaria indica (SiPMEI, XM_004978185.1); Arabidopsis thaliana (AtINH, Y12807; AtINVINH1, DQ056716); Solanum tuberosum (StINVINH2α, StINVINH2α, KJ788176; StINVINH2αB, FJ810207; StINVINH2αC, FJ810208; StINVINH2αD, FJ810209; StINVINH, JQ269669; StINVINH2B, GU321342), and Nicotiana tabacum (NtVINVINH, AY145781; NtINVINH, AY594179; NtINVINH2, Y12805; NtINVINH1, Y12806). Values indicate % similarity of INVINH proteins against each other. Colored boxes with numbers indicate degree of similarity: Light to dark red indicates moderate to high similarity whereas light to dark blue indicates moderately low to least similarity. [file Image_7.PDF]

| | | 1 | 2 | 3 | 4 | 5 | 6 | 7 | 8 | 9 | 10 | 11 | 12 | 13 | 14 | 15 | 16 | 17 | 18 | 19 | 20 | 21 | 22 | 23 | 24 | 25 | 26 | 27 | 28 | 29 | 30 | 31 | 32 | 33 | 34 |
|---|---|---|---|---|---|---|---|---|---|---|---|---|---|---|---|---|---|---|---|---|---|---|---|---|---|---|---|---|---|---|---|---|---|---|---|
| Ssine | 1 | | 96.86 | 98.95 | 93.33 | 81.60 | 80.83 | 84.97 | 64.58 | 43.08 | 43.08 | 43.59 | 43.08 | 43.81 | 19.71 | 20.49 | 20.49 | 22.63 | 16.44 | 18.80 | 15.32 | 15.32 | 15.32 | 15.32 | 15.38 | 15.98 | 15.53 | 15.91 | 13.55 | 15.28 | 15.28 | 15.28 | 15.49 | 20.47 | 19.62 |
| SolNH | 2 | 96.86 | | 95.81 | 95.79 | 78.77 | 77.72 | 82.90 | 63.68 | 44.21 | 44.21 | 44.74 | 44.21 | 44.97 | 20.20 | 20.79 | 20.79 | 23.11 | 16.82 | 19.13 | 15.67 | 15.67 | 15.67 | 15.67 | 15.74 | 16.36 | 15.89 | 16.28 | 13.88 | 15.64 | 15.64 | 15.64 | 15.87 | 20.09 | 20.00 |
| ShINH2 | 3 | 98.95 | 95.81 | | 92.31 | 82.08 | 79.79 | 84.97 | 65.10 | 43.08 | 43.08 | 43.59 | 43.08 | 43.81 | 19.71 | 20.49 | 20.49 | 22.63 | 16.44 | 18.80 | 15.32 | 15.32 | 15.32 | 15.32 | 15.38 | 15.98 | 15.53 | 15.91 | 13.55 | 15.28 | 15.28 | 15.28 | 15.49 | 20.93 | 19.62 |
| SbarINH | 4 | 93.33 | 95.79 | 92.31 | | 75.93 | 74.62 | 81.22 | 61.86 | 43.01 | 43.01 | 43.52 | 43.01 | 43.75 | 19.81 | 20.39 | 20.39 | 22.41 | 16.52 | 18.38 | 15.45 | 15.45 | 15.45 | 15.45 | 15.53 | 16.13 | 15.67 | 16.06 | 13.68 | 15.42 | 15.42 | 15.42 | 15.64 | 19.72 | 19.77 |
| SspoINH | 5 | 81.60 | 78.77 | 82.08 | 75.93 | | 64.49 | 71.96 | 53.30 | 34.88 | 34.88 | 35.35 | 34.42 | 35.51 | 15.91 | 16.67 | 16.67 | 21.49 | 15.18 | 16.13 | 12.92 | 12.92 | 12.92 | 12.92 | 12.97 | 13.50 | 13.08 | 13.45 | 11.21 | 13.30 | 13.30 | 13.30 | 13.22 | 14.96 | 17.74 |
| SrINH | 6 | 80.83 | 77.72 | 79.79 | 74.62 | 64.49 | | 68.21 | 49.48 | 34.69 | 34.69 | 35.20 | 35.71 | 35.38 | 18.75 | 15.53 | 15.53 | 17.14 | 15.11 | 14.10 | 10.76 | 10.76 | 10.76 | 10.76 | 10.81 | 11.36 | 10.91 | 11.31 | 11.57 | 10.60 | 10.60 | 10.60 | 14.49 | 18.52 | 15.47 |
| ZmINVINH | 7 | 84.97 | 82.90 | 84.97 | 81.22 | 71.96 | 68.21 | | 63.73 | 44.10 | 44.10 | 44.62 | 44.10 | 43.81 | 19.23 | 20.98 | 20.98 | 20.58 | 18.58 | 19.23 | 16.67 | 16.67 | 16.67 | 16.67 | 16.74 | 17.35 | 16.89 | 17.27 | 14.95 | 16.20 | 16.20 | 16.20 | 15.49 | 20.28 | 19.25 |
| AegtauINH | 8 | 64.58 | 63.68 | 65.10 | 61.86 | 53.30 | 49.48 | 63.73 | | 48.96 | 47.92 | 49.48 | 47.92 | 47.12 | 23.27 | 20.69 | 20.69 | 22.59 | 17.41 | 18.10 | 13.18 | 13.18 | 13.18 | 13.18 | 13.24 | 13.82 | 13.36 | 13.76 | 10.85 | 14.49 | 14.49 | 14.49 | 15.17 | 18.98 | 18.39 |
| ShINH1 | 9 | 43.08 | 44.21 | 43.08 | 43.01 | 34.88 | 34.69 | 44.10 | 48.96 | | 97.18 | 92.70 | 85.88 | 74.72 | 23.83 | 21.65 | 21.65 | 21.30 | 19.16 | 16.07 | 18.10 | 18.10 | 18.10 | 18.10 | 18.18 | 18.84 | 18.84 | 18.75 | 14.36 | 18.14 | 18.14 | 18.14 | 15.92 | 17.14 | 17.13 |
| SoInvinh1 | 10 | 43.08 | 44.21 | 43.08 | 43.01 | 34.88 | 34.69 | 44.10 | 47.92 | 97.18 | | 92.09 | 86.36 | 74.58 | 22.80 | 21.24 | 21.24 | 21.83 | 19.72 | 15.70 | 18.18 | 18.18 | 18.18 | 18.18 | 18.27 | 18.93 | 18.93 | 18.84 | 14.43 | 17.73 | 17.73 | 17.73 | 16.50 | 17.22 | 17.60 |
| SbINHCWI/VAI | 11 | 43.59 | 44.74 | 43.59 | 43.52 | 35.35 | 35.20 | 44.62 | 49.48 | 92.70 | 92.09 | | 87.57 | 75.14 | 24.35 | 21.24 | 21.24 | 20.52 | 20.09 | 16.14 | 17.70 | 17.70 | 17.70 | 17.70 | 17.79 | 18.45 | 18.45 | 18.36 | 13.93 | 16.75 | 16.75 | 16.75 | 15.50 | 17.70 | 16.80 |
| ZmINHCWI/VAI | 12 | 43.08 | 44.21 | 43.08 | 43.01 | 34.42 | 35.71 | 44.10 | 47.92 | 85.88 | 86.36 | 87.57 | | 73.45 | 23.32 | 21.76 | 21.76 | 20.09 | 19.25 | 14.80 | 17.70 | 17.70 | 17.70 | 17.70 | 17.79 | 18.45 | 18.45 | 18.36 | 14.93 | 16.75 | 16.75 | 16.75 | 14.00 | 17.22 | 16.00 |
| SiPMEI | 13 | 43.81 | 44.97 | 43.81 | 43.75 | 35.51 | 35.38 | 43.81 | 47.12 | 74.72 | 74.58 | 75.14 | 73.45 | | 20.21 | 20.83 | 20.83 | 18.42 | 15.89 | 15.25 | 17.70 | 17.70 | 17.70 | 17.70 | 17.79 | 17.48 | 17.48 | 17.39 | 13.43 | 16.75 | 16.75 | 16.75 | 14.00 | 17.22 | 16.06 |
| AtINH | 14 | 19.71 | 20.20 | 19.71 | 19.81 | 15.91 | 18.75 | 19.23 | 23.27 | 23.83 | 22.80 | 24.35 | 23.32 | 20.21 | | 16.08 | 16.08 | 14.29 | 13.24 | 12.61 | 12.86 | 12.86 | 12.86 | 12.86 | 12.92 | 13.04 | 13.53 | 12.50 | 11.71 | 13.66 | 13.66 | 13.66 | 15.42 | 15.64 | 14.46 |
| ZmINVINH1 | 15 | 20.49 | 20.79 | 20.49 | 20.39 | 16.67 | 15.53 | 20.98 | 20.69 | 21.65 | 21.24 | 21.24 | 21.76 | 20.83 | 16.08 | | 100.00 | 16.10 | 11.82 | 13.22 | 16.74 | 16.74 | 16.74 | 16.74 | 16.82 | 17.45 | 17.45 | 17.37 | 15.46 | 17.14 | 17.14 | 17.14 | 16.02 | 13.49 | 14.23 |
| ZmINVINH2 | 16 | 20.49 | 20.79 | 20.49 | 20.39 | 16.67 | 15.53 | 20.98 | 20.69 | 21.65 | 21.24 | 21.24 | 21.76 | 20.83 | 16.08 | 100.00 | | 16.10 | 11.82 | 13.22 | 16.74 | 16.74 | 16.74 | 16.74 | 16.82 | 17.45 | 17.45 | 17.37 | 15.46 | 17.14 | 17.14 | 17.14 | 16.02 | 13.49 | 14.23 |
| SbINH | 17 | 22.63 | 23.11 | 22.63 | 22.41 | 21.49 | 17.14 | 20.58 | 22.59 | 21.30 | 21.83 | 20.52 | 20.09 | 18.42 | 14.29 | 16.10 | 16.10 | | 15.32 | 14.29 | 13.93 | 13.93 | 13.93 | 13.93 | 13.93 | 13.93 | 13.52 | 14.34 | 12.55 | 14.75 | 14.75 | 14.75 | 13.11 | 13.52 | 16.33 |
| AegtauINH1 | 18 | 16.44 | 16.82 | 16.44 | 16.52 | 15.18 | 15.11 | 18.58 | 17.41 | 19.16 | 19.72 | 20.09 | 19.25 | 15.89 | 13.24 | 11.82 | 11.82 | 15.32 | | 6.53 | 9.32 | 9.32 | 9.32 | 9.32 | 9.36 | 9.44 | 9.44 | 9.83 | 8.30 | 8.26 | 8.26 | 8.26 | 8.81 | 12.93 | 11.81 |
| AtINVINH1 | 19 | 18.80 | 19.13 | 18.80 | 18.38 | 16.13 | 14.10 | 19.23 | 18.10 | 16.07 | 15.70 | 16.14 | 14.80 | 15.25 | 12.61 | 13.22 | 13.22 | 14.29 | 6.53 | | 10.81 | 10.81 | 10.81 | 10.81 | 10.81 | 10.81 | 10.81 | 10.36 | 9.50 | 9.50 | 9.50 | 9.50 | 11.71 | 11.01 | 10.42 |
| StINVINH2alphaA1 | 20 | 15.32 | 15.67 | 15.32 | 15.45 | 12.92 | 10.76 | 16.67 | 13.18 | 18.10 | 18.18 | 17.70 | 17.70 | 17.70 | 12.86 | 16.74 | 16.74 | 13.93 | 9.32 | 10.81 | | 100.00 | 100.00 | 100.00 | 98.90 | 97.79 | 96.69 | 96.69 | 80.66 | 70.72 | 70.72 | 70.72 | 43.17 | 15.69 | 13.04 |
| StINVINH2alphaA2 | 21 | 15.32 | 15.67 | 15.32 | 15.45 | 12.92 | 10.76 | 16.67 | 13.18 | 18.10 | 18.18 | 17.70 | 17.70 | 17.70 | 12.86 | 16.74 | 16.74 | 13.93 | 9.32 | 10.81 | 100.00 | | 100.00 | 100.00 | 98.90 | 97.79 | 96.69 | 96.69 | 80.66 | 70.72 | 70.72 | 70.72 | 43.17 | 15.69 | 13.04 |
| StINVINH2A | 22 | 15.32 | 15.67 | 15.32 | 15.45 | 12.92 | 10.76 | 16.67 | 13.18 | 18.10 | 18.18 | 17.70 | 17.70 | 17.70 | 12.86 | 16.74 | 16.74 | 13.93 | 9.32 | 10.81 | 100.00 | 100.00 | | 100.00 | 98.90 | 97.79 | 96.69 | 96.69 | 80.66 | 70.72 | 70.72 | 70.72 | 43.17 | 15.69 | 13.04 |
| StINVINH2alpha | 23 | 15.32 | 15.67 | 15.32 | 15.45 | 12.92 | 10.76 | 16.67 | 13.18 | 18.10 | 18.18 | 17.70 | 17.70 | 17.70 | 12.86 | 16.74 | 16.74 | 13.93 | 9.32 | 10.81 | 100.00 | 100.00 | 100.00 | | 98.90 | 97.79 | 96.69 | 96.69 | 80.66 | 70.72 | 70.72 | 70.72 | 43.17 | 15.69 | 13.04 |
| StINVINH2alphaB | 24 | 15.38 | 15.74 | 15.38 | 15.53 | 12.97 | 10.81 | 16.74 | 13.24 | 18.18 | 18.27 | 17.79 | 17.79 | 17.79 | 12.92 | 16.82 | 16.82 | 13.93 | 9.36 | 10.81 | 98.90 | 98.90 | 98.90 | 98.90 | | 97.78 | 96.67 | 96.67 | 81.11 | 71.11 | 71.11 | 71.11 | 43.41 | 15.76 | 13.04 |
| StINVINH2alphaC | 25 | 15.98 | 16.36 | 15.98 | 16.13 | 13.50 | 11.36 | 17.35 | 13.82 | 18.84 | 18.93 | 18.45 | 18.45 | 17.48 | 13.04 | 17.45 | 17.45 | 13.93 | 9.44 | 10.81 | 97.79 | 97.79 | 97.79 | 97.79 | 97.78 | | 98.88 | 96.67 | 82.58 | 72.47 | 72.47 | 72.47 | 44.44 | 15.92 | 13.04 |
| StINVINH2alphaD | 26 | 15.53 | 15.89 | 15.53 | 15.67 | 13.08 | 10.91 | 16.89 | 13.36 | 18.84 | 18.93 | 18.45 | 18.45 | 17.48 | 13.53 | 17.45 | 17.45 | 13.52 | 9.44 | 10.81 | 96.69 | 96.69 | 96.69 | 96.69 | 96.67 | 98.88 | | 95.56 | 81.46 | 71.91 | 71.91 | 71.91 | 44.44 | 15.42 | 12.65 |
| StINVINH | 27 | 15.91 | 16.28 | 15.91 | 16.06 | 13.45 | 11.31 | 17.27 | 13.76 | 18.75 | 18.84 | 18.36 | 18.36 | 17.39 | 12.50 | 17.37 | 17.37 | 14.34 | 9.83 | 10.36 | 96.69 | 96.69 | 96.69 | 96.69 | 96.67 | 96.67 | 95.56 | | 79.44 | 72.63 | 72.63 | 72.63 | 43.65 | 15.84 | 12.65 |
| StINVINH2B | 28 | 13.55 | 13.88 | 13.55 | 13.68 | 11.21 | 11.57 | 14.95 | 10.85 | 14.36 | 14.43 | 13.93 | 14.93 | 13.43 | 11.71 | 15.46 | 15.46 | 12.55 | 8.30 | 9.50 | 80.66 | 80.66 | 80.66 | 80.66 | 81.11 | 82.58 | 81.46 | 79.44 | | 61.58 | 61.58 | 61.58 | 39.55 | 13.43 | 11.16 |
| NtVINVINH | 29 | 15.28 | 15.64 | 15.28 | 15.42 | 13.30 | 10.60 | 16.20 | 14.49 | 18.14 | 17.73 | 16.75 | 16.75 | 16.75 | 13.66 | 17.14 | 17.14 | 14.75 | 8.26 | 9.50 | 70.72 | 70.72 | 70.72 | 70.72 | 71.11 | 72.47 | 71.91 | 72.63 | 61.58 | | 100.00 | 100.00 | 43.18 | 16.08 | 13.44 |
| NtINVINH | 30 | 15.28 | 15.64 | 15.28 | 15.42 | 13.30 | 10.60 | 16.20 | 14.49 | 18.14 | 17.73 | 16.75 | 16.75 | 16.75 | 13.66 | 17.14 | 17.14 | 14.75 | 8.26 | 9.50 | 70.72 | 70.72 | 70.72 | 70.72 | 71.11 | 72.47 | 71.91 | 72.63 | 61.58 | 100.00 | | 100.00 | 43.18 | 16.08 | 13.44 |
| NtINVINH2 | 31 | 15.28 | 15.64 | 15.28 | 15.42 | 13.30 | 10.60 | 16.20 | 14.49 | 18.14 | 17.73 | 16.75 | 16.75 | 16.75 | 13.66 | 17.14 | 17.14 | 14.75 | 8.26 | 9.50 | 70.72 | 70.72 | 70.72 | 70.72 | 71.11 | 72.47 | 71.91 | 72.63 | 61.58 | 100.00 | 100.00 | | 43.18 | 16.08 | 13.44 |
| NtINVINH1 | 32 | 15.49 | 15.87 | 15.49 | 15.64 | 13.22 | 14.49 | 15.49 | 15.17 | 15.92 | 16.50 | 15.50 | 14.00 | 14.00 | 15.42 | 16.02 | 16.02 | 13.11 | 8.81 | 11.71 | 43.17 | 43.17 | 43.17 | 43.17 | 43.41 | 44.44 | 44.44 | 43.65 | 39.55 | 43.18 | 43.18 | 43.18 | | 16.75 | 11.46 |
| SbINH2 | 33 | 20.47 | 20.09 | 20.93 | 19.72 | 14.96 | 18.52 | 20.28 | 18.98 | 17.14 | 17.22 | 17.70 | 17.22 | 17.22 | 15.64 | 13.49 | 13.49 | 13.52 | 12.93 | 11.01 | 15.69 | 15.69 | 15.69 | 15.69 | 15.76 | 15.92 | 15.42 | 15.84 | 13.43 | 16.08 | 43.18 | 16.08 | 16.75 | | 13.13 |
| SbINH1 | 34 | 19.62 | 20.00 | 19.62 | 19.77 | 17.74 | 15.47 | 19.25 | 18.39 | 17.13 | 17.60 | 16.80 | 16.00 | 16.06 | 14.46 | 14.23 | 14.23 | 16.33 | 11.81 | 10.42 | 13.04 | 13.04 | 13.04 | 13.04 | 13.04 | 13.04 | 12.65 | 12.65 | 11.16 | 13.44 | 13.44 | 13.44 | 11.46 | 13.13 | |
