# Supplementary material for: Identification and Functional Characterization of Sugarcane Invertase Inhibitor (ShINH1): A Potential Candidate for Reducing Pre- and Post-harvest Loss of Sucrose in Sugarcane
Source: Front Plant Sci. 2018 May 3;9:598. doi: 10.3389/fpls.2018.00598 (PMC5944049; doi:10.3389/fpls.2018.00598)
Supplement: Figure S8 — Prediction of secondary structure of ShINH1 and ShINH2 proteins. Deduced amino acid sequences of ShINH1 and ShINH2 proteins were analyzed for secondary structure using PSIPRED tool (http://bioinf.cs.ucl.ac.uk/psipred/). Both (A) ShINH1 and (B) ShINH2 were predicted to form predominantly α-helical secondary structure. Helical structure is indicated by the letter “H” and pink cylinders whereas, coil regions are indicated by the letter “C” and horizontal lines. Confidence of prediction is shown by the blue colored bars. [file Image_8.PDF]

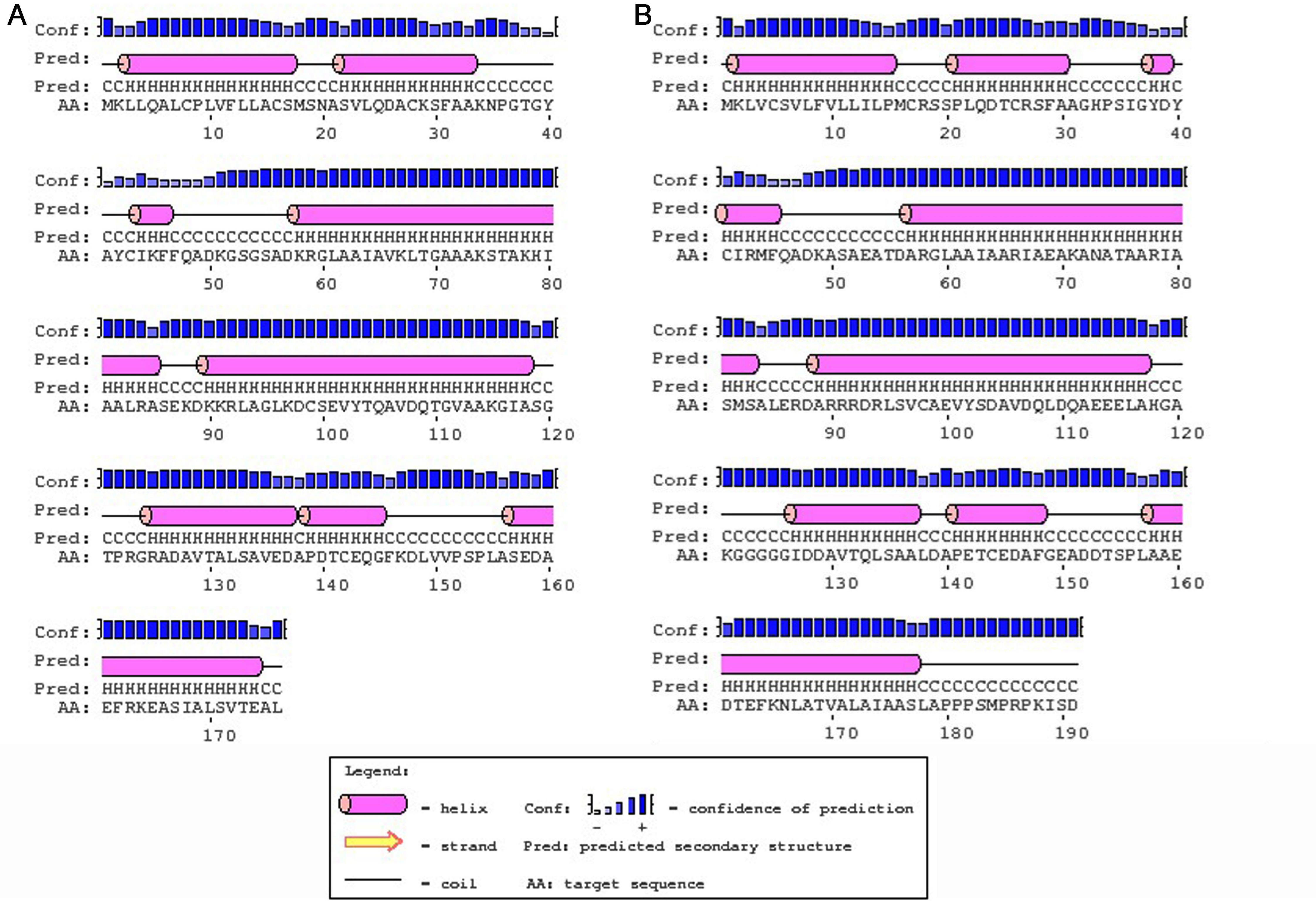
A
Pred: CCHHHHHHHHHHHHHHHHCCCCHHHHHHHHHHHHHCCCCCCC
AA: MKLLQALCPLVFLLACSMSNASVLQDACKSFAAKNPGTGY
Pred: CCHHHCCCCCCCCCCCHHHHHHHHHHHHHHHHHHHHHHHH
AA: AYCIKFFQADKGSGSADKRGLAAIAVKLTGAAAKSTAKHI
Pred: HHHHHCCCCHHHHHHHHHHHHHHHHHHHHHHHHHHHHHCC
AA: AALRASEKDKKRLAGLKDCSEVYTQAVDQTGVAAKGIASG
Pred: CCCCHHHHHHHHHHHHHHCHHHHHHHCCCCCCCCCCHHHH
AA: TPRGRADAVTALSAVEDAPDTCEQGFKDLVVPSPLASEDA
Pred: HHHHHHHHHHHHHHHCC
AA: EFRKEASIALSVTEAL
B
Pred: CHHHHHHHHHHHHHHHCCCCCHHHHHHHHHHHCCCCCCCHHC
AA: MKLVCSVLFVLLILPMCRSSPLQDTCRSFAAGHPSIGYDY
Pred: HHHHHCCCCCCCCCCCHHHHHHHHHHHHHHHHHHHHHHHH
AA: CIRMFQADKASAEATDARGLAAIAARIAEAKANATAARIA
Pred: HHHCCCCCHHHHHHHHHHHHHHHHHHHHHHHHHHHHHCCC
AA: SMSALERDARRRDRLSVCAEVYSDAVDQLDQAEEELAHGA
Pred: CCCCCCHHHHHHHHHHHCCCHHHHHHHHCCCCCCCCCHHH
AA: KGGGGGIDDAVTQLSAALDAPETCEDAFGEADDTSPLAAE
Pred: HHHHHHHHHHHHHHHHHHCCCCCCCCCCCCCC
AA: DTEFKNLATVALAIAASLAPPPSMPRPKISD
Legend:
- helix
- strand
- coil
Conf: - confidence of prediction
Pred: predicted secondary structure
AA: target sequence
